# Identification of Predictors of Metastatic Potential in Paragangliomas to Develop a Prognostic Score (PSPGL)

**DOI:** 10.1210/jendso/bvae093

**Published:** 2024-05-07

**Authors:** Daniela Yone Veiga Iguchi, Sebastião Nunes Martins Filho, Iberê Cauduro Soares, Sheila Aparecida Coelho Siqueira, Venâncio Avancini Ferreira Alves, Aline Kawassaki Assato, Ji Hoon Yang, Madson Q Almeida, Maria Candida Barisson Villares Fragoso, Gustavo Freitas Cardoso Fagundes, Berenice B Mendonca, Delmar Muniz Lourenço Junior, Ana O Hoff, Luciana Audi Castroneves, Bruno Ferraz-de-Souza, Maria Lucia Cardillo Correa Giannella, Maria Adelaide Albergaria Pereira

**Affiliations:** Divisão de Endocrinologia e Metabologia, Hospital das Clínicas, Faculdade de Medicina da Universidade de São Paulo, São Paulo 05403-000, Brazil; Department of Laboratory Medicine and Pathology, University of Alberta, Alberta T6G 1C9, Canada; Divisão de Anatomia Patológica, Instituto do Câncer do Estado de São Paulo (ICESP), Faculdade de Medicina da Universidade de São Paulo, São Paulo 01246-000, Brazil; Divisão de Anatomia Patológica, Hospital das Clínicas, Faculdade de Medicina da Universidade de São Paulo, São Paulo 05403-000, Brazil; Laboratório de Investigaçãoc Médica LIM/14, Departamento de Patologia, Faculdade de Medicina da Universidade de São Paulo, São Paulo 01246-903, Brazil; Laboratório de Investigaçãoc Médica LIM/14, Departamento de Patologia, Faculdade de Medicina da Universidade de São Paulo, São Paulo 01246-903, Brazil; Clínica de Endocrinologia, Hospital do Servidor Público Municipal de São Paulo, São Paulo 01532-000, Brazil; Laboratório de Endocrinologia Molecular e Celular LIM/25, Divisão de Endocrinologia e Metabologia, Hospital das Clínicas, Faculdade de Medicina da Universidade de São Paulo, São Paulo 01246-903, Brazil; Laboratório de Hormônios e Genética Molecular LIM/42, Divisão de Endocrinologia e Metabologia, Hospital das Clínicas, Faculdade de Medicina da Universidade de São Paulo, São Paulo 01246-903, Brazil; Divisão de Endocrinologia e Metabologia, Instituto do Câncer do Estado de São Paulo (ICESP), Faculdade de Medicina da Universidade de São Paulo, São Paulo 01246-000, Brazil; Laboratório de Endocrinologia Molecular e Celular LIM/25, Divisão de Endocrinologia e Metabologia, Hospital das Clínicas, Faculdade de Medicina da Universidade de São Paulo, São Paulo 01246-903, Brazil; Laboratório de Hormônios e Genética Molecular LIM/42, Laboratório de Sequenciamento em Larga Escala (SELA), Divisão de Endocrinologia e Metabologia, Hospital das Clínicas, Faculdade de Medicina da Universidade de São Paulo, São Paulo 01246-903, Brazil; Laboratório de Endocrinologia Molecular e Celular LIM/25, Divisão de Endocrinologia e Metabologia, Hospital das Clínicas, Faculdade de Medicina da Universidade de São Paulo, São Paulo 01246-903, Brazil; Divisão de Endocrinologia e Metabologia, Instituto do Câncer do Estado de São Paulo (ICESP), Faculdade de Medicina da Universidade de São Paulo, São Paulo 01246-000, Brazil; Divisão de Endocrinologia e Metabologia, Instituto do Câncer do Estado de São Paulo (ICESP), Faculdade de Medicina da Universidade de São Paulo, São Paulo 01246-000, Brazil; Laboratório de Endocrinologia Celular e Molecular LIM/25, Divisão de Endocrinologia e Metabologia, Hospital das Clínicas, Faculdade de Medicina da Universidade de São Paulo 01246-903, Brazil; School of Medicine, University of Notre Dame Australia, Fremantle WA 6160, Australia; Laboratório de Carboidratos e Radioimunoensaio LIM/18, Divisão de Endocrinologia e Metabologia, Hospital das Clínicas, Faculdade de Medicina da Universidade de São Paulo, São Paulo 01246-903, Brazil; Divisão de Endocrinologia e Metabologia, Hospital das Clínicas, Faculdade de Medicina da Universidade de São Paulo, São Paulo 05403-000, Brazil

**Keywords:** paraganglioma, pheochromocytoma, metastatic paraganglioma, non-metastatic paraganglioma, PASS, GAPP

## Abstract

**Context:**

Paragangliomas (PGLs) are rare tumors in adrenal and extra-adrenal locations. Metastasis are found in approximately 5% to 35% of PGLs, and there are no reliable predictors of metastatic disease.

**Objective:**

This work aimed to develop a prognostic score of metastatic potential in PGLs.

**Methods:**

A retrospective analysis was conducted of clinical data from a cohort with PGLs and tumor histological assessment. Patients were divided into metastatic PGL (presence of metastasis) and nonmetastatic PGL (absence of metastasis ≥96 months of follow-up) groups. Univariate and multivariable analysis were performed to identify predictors of metastatic potential. A prognostic score was developed based on coefficients of multivariable analysis. Kaplan-Meier curves were generated to estimate disease-specific survival (DSS).

**Results:**

Out of 263 patients, 35 patients had metastatic PGL and 110 patients had nonmetastatic PGL. In multivariable analysis, 4 features were independently related to metastatic disease and composed the Prognostic Score of Paragangliomas (PSPGL): presence of central or confluent necrosis (33 points), more than 3 mitosis/10 high-power field (HPF) (28 points), extension into adipose tissue (20 points), and extra-adrenal location (19 points). A PSPGL of 24 or greater showed similar sensitivity with higher specificity than the Pheochromocytoma of the Adrenal Gland Scaled Score (PASS) and Grading System for Adrenal Pheochromocytoma and Paraganglioma (GAPP). PSPGL less than or equal to 20 was associated with a risk of metastasis of approximately 10%, whereas a PSPGL of 40 or greater was associated with approximately 80%. The presence of metastasis and Ki-67 of 3% or greater were related to lower DSS.

**Conclusion:**

The PSPGL, composed of 4 easy-to-assess parameters, demonstrated good performance in predicting metastatic potential and good ability in estimating metastasis risk.

Paragangliomas (PGLs) are rare tumors located in the adrenal medulla (adrenal paragangliomas - PGLAd), previously known as pheochromocytomas, or in extra-adrenal locations (extra-adrenal paragangliomas - PGLexAd) [1-4]. Metastatic disease is defined as the presence of chromaffin tissue in nonchromaffin organs [5], it is reported in 5% to 20% of PGLAd and in 15% to 35% of PGLexAd [6-9], and early diagnosis is important for a better treatment response [10-13]. Metastasis can be present at the time of diagnosis or can develop many years after primary tumor surgery, requiring patient long-term surveillance after surgery [8, 9, 14, 15]. Therefore, determining the metastatic potential of these tumors is of great interest as it would allow for the individualization of follow-up for different patient profiles after primary tumor surgery.

Some characteristics of patients and of tumors have been suggested as predictors of metastatic behavior. Of note are, a younger age at diagnosis [16-19], the type of catecholamine secreted by the tumor [20-22], the location [12, 19, 22-24], and tumor size [12, 16, 19, 22, 24, 25]. The presence of a germline pathogenic variant (PV) in the gene encoding the subunit B of succinate dehydrogenase (SDHB) is a well-established risk factor found in 30% to 50% of patients with metastatic PGL [18, 26-31].

As none of these criteria have, alone, sufficient sensitivity and specificity for predicting metastatic potential, scores have been developed. These scores are composed of histological and nonhistological parameters that when used together allow the risk of metastasis in PGLs to be estimated. The Pheochromocytoma of the Adrenal Gland Scaled Score (PASS) proposed by Thompson in 2002 [32] and the Grading System for Adrenal Phaeochromocytoma and Paraganglioma (GAPP) proposed by Kimura and colleagues in 2005 [33] are the best-known scoring systems. PASS, composed of 12 histological parameters, has been validated in several studies [17, 34-38], but problems related to its reproducibility and conflicting data about its specificity in identifying metastatic potential in PGL do not allow for this score to be used as the only tool to predict future behavior of these tumors [39-41]. GAPP combined some of histological parameters included in PASS with immunohistochemical (IHC) and biochemical tumor characteristics and was later expanded with the participation of several centers in Japan [42]. Recently a study demonstrated advantages of GAPP over PASS regarding prediction of metastatic behavior and reproducibility [40]. Other prognostic scores include the modified GAPP score (M-GAPP) [36], the Composite Pheochromocytoma/Paraganglioma Prognostic Score (COPPS) [37], and the Age, Size, Extra-adrenal location, Secretory type (ASES) score [43].

Some IHC and molecular markers have been studied as predictors of metastatic behavior in PGLs showing varying levels of evidence. The classic IHC marker to predict malignancy is Ki-67 [23, 33, 44-47] but others have already been suggested, such as zinc-finger transcription factor SNAIL, signal transducers and activators of transcription 3 (STAT3), human antigen R (HuR), cyclooxygenase-2 (COX-2), vascular endothelial growth factor (VEGF), hypoxia inducible factor 1a (HIF-1a), and somatostatin receptors type 2 (SSTR2) [48-54]. Molecular assessment of tumor tissue suggests somatic pathogenic variants in the genes mastermind like transcriptional coactivator 3 (*MAML3*), alpha thalassemia/mental retardation syndrome X (*ATRX*), and cold shock domain-containing E1 (*CSDE1*) are also associated with metastatic behavior [55-57]. The expression of human telomerase reverse transcriptase (hTERT) and heat shock protein 90 (HSP90) is higher in metastatic tumors [58]. Other markers such as chromogranin B and S100 protein have been associated with nonmetastatic tumor behavior [23, 38, 59-63].

Cocaine- and amphetamine-regulated transcript (CART) is a highly expressed peptide in the rat brain in response to psychostimulants [64] and is poorly studied for predicting the metastatic potential of PGLs. Studies that assessed plasma concentrations of CART in patients with PGLs have shown that elevated levels of this peptide correlate positively with disease progression [65, 66]. No studies have assessed the performance of CART as an IHC marker in predicting metastatic potential in PGLs.

The objective of this investigation was to identify predictors of metastatic potential in PGLs and select the best predictors to compose the Prognostic Score of Paragangliomas (PSPGL). In addition, we investigated factors related to worse prognosis in patients with metastatic disease.

## Materials and Methods

### Ethical Considerations

The research protocol was approved by the local ethics in research commission (Comissão de Ética para Análise de Projetos de Pesquisa do HCFMUSP—CAPPESQ, consubstantiated opinion No. 4.920.314).

### Population

Participants included patients diagnosed with PGL and followed at a single center (Hospital das Clínicas da Faculdade de Medicina da Universidade de São Paulo—HC-FMUSP, São Paulo, Brazil), from 1967 to 2019. Clinical, laboratorial, and genetic data from patients were obtained from medical records and were retrospectively analyzed; the histological and IHC data of tumors were newly reviewed. Patients admitted until 2019 for whom we had access to progression data up to July 2023, were included in the study.

### Clinical Data

Data were retrospectively collected from medical records and included age, sex, clinical presentation at initial diagnosis (presence and duration of signs and symptoms or incidentaloma or genetic screening), follow-up time between diagnosis and last assessment or death, absence or presence of metastasis including time and site of appearance, and data related to genetic, biochemical, and topographic diagnosis. The genetic diagnosis was clinical (family history of PGL or of other tumors related to syndromic genetic diseases such as multiple endocrine neoplasia type 2 and von Hippel-Lindau disease [VHL]), and/or molecular as of when this technique became available. Molecular genetic investigations, conducted in DNA extracted from peripheral blood leukocytes, were performed, initially, using Sanger method (*VHL*, succinate dehydrogenase complex subunits [*SDHB*, *SDHC*, *SDHD*], myc-associated factor X [*MAX*], transmembrane protein 127 [*TMEM127*]). In patients without a genetic diagnosis defined by this method, multiplex ligation-dependent probe amplification (MLPA—*SDHx* and *VHL*) was performed. Patients who remained without genetic diagnosis after using both methods were investigated using a target next-generation sequencing panel on Illumina NextSeq 500 platform sequencers (Illumina Inc) that includes the following genes: fumarate hydratase (*FH*), *MAX*, neurofibromatosis 1 (*NF1*), rearranged during transfection (*RET*), succinate dehydrogenase complex subunit (*SDHA*, *SDHB*, *SDHC*, *SDHD*), *TMEM127*, *VHL*, Egl-9 family hypoxia-inducible factor 1 (*ENGL-1*), endothelial PAS domain protein 1 (*EPAS1*), kinesin family member *1B* (*KIF1B*), proto-oncogene, receptor tyrosine kinase (*MET*), succinate dehydrogenase complex assembly factor 2 (*SDHAF2*), ATRX chromatin remodeler (*ATRX*), B-Raf proto-oncogene- serine/threonine kinase (*BRAF*), fibroblast growth factor receptor 1 (*FGFR1*), HRas proto-oncogene-GTPase (*HRAS*), lysine methyltransferase 2D (*KMT2D*), and cellular tumor antigen P53 (*P53*) [55, 67-72]. Biochemical diagnosis and tumor functionality were assessed by determining catecholamines and/or their metabolites in 24-hour urine (U) or in plasma (P): vanilmandelic acid (VMAU), total metanephrines (tMnU), fractionated catecholamines (adrenaline [AU and AP], noradrenaline [NAU and NAP], and dopamine [DopaU and DopaP]), and free and fractionated metanephrines (metanephrines [MnU and MnP] and normetanephrines [NMnU and NMnP]). Tumors were classified as functional or nonfunctional based on these determinations and when biochemical evaluation data were unavailable tumors were classified by the presence or absence of typical PGL clinical presentation. Functional tumors were classified as adrenergic (increased concentrations of adrenaline or its metabolites regardless of noradrenaline and/or its metabolites or dopamine concentrations) and noradrenergic (increased concentrations of noradrenaline or its metabolites with adrenaline and/or its metabolites within the normal reference range). Data regarding topographic diagnosis of the tumor were collected from the following imaging exams: abdominal ultrasound, computed tomography (CT), magnetic resonance imaging, and 123/131-metaiodobenzylguanidine (^123/131^MIBG). For investigation of metastases, in addition to these methods, ^111^In-pentetreotida scintigraphy scan (OctreoScan), 18-fluordeoxyglucose positron emission tomography scan (**^18^**F-FDG PET/CT), and 68-gallium DOTATATE PET scan (**^68^**Ga-DOTATE PET/CT) were performed in some patients. Tumor size was obtained from macroscopic analysis of the tumor after surgery or by analyzing presurgical imaging exams.

The methods used in the biochemical analyses were capillary electrophoresis with spectrophotometric detection (VMAU), Pisano ion-exchange chromatography (tMnU), high-performance liquid chromatography (HPLC) with electrochemical detection (AU, AP, NAU, NAP, DopaU, DopaP, MnU, and NMnU), and liquid chromatography with tandem mass spectrometry (LC-MS/MS—MnP and NMnP).

### Histology

Slides of each tumor, stained with hematoxylin and eosin (HE), were provided by the Department of Pathological Anatomy of HC-FMUSP and were reviewed by only one pathologist (pathologist 1) with experience in adrenal pathology and who was blinded to clinical data. In the cases of patients with multiple tumors, one tumor per patient was considered, opting for the tumor with the larger size and/or higher PASS score.

### Immunohistochemistry

IHC evaluation was performed by 2 pathologists (1 and 2). All the IHC studies carried out used paraffine sections (4 µm for IHC-Ki-67 and 3 µm for synaptophysin, chromogranin A [CHGA], chromogranin B [CHGB], and CART) for slide preparation. The slides were deparaffinized and rehydrated before IHC reactions were carried out. In the IHC–Ki-67 study, the slides were immersed in citrate buffer solution pH 6.0 at 95 °C and steamed-treated for 40 minutes for antigen retrieval. After peroxidase blocking, the slides were incubated with the primary antibody (mouse monoclonal antibody MIB1, 1:100 dilution, DAKO, RRID: AB_2142367) for 18 to 24 hours at 4 °C. Signal amplification was performed using the Novolink Polymer Detection System (Vision Biosystems), followed by diaminobenzidine tetrahydrochloride and dimethyl sulfoxide (DAB) reaction (Sigma). The slides were stained with hematoxylin and covered with Entellan (Merck). Nontumoral lymph node slides were used as an external control for the reaction, and intratumoral lymphocytes were used as an internal control. The slides were scanned using a Pannoramic 250 Flash III scanner with Pannoramic Viewer 1:15 software (3DHISTECH). Assessment of the Ki-67 index was performed by automatic counting of tumor hot spots using QuPath software [73]. The areas were selected by pathologist 1, who assessed at least 500 cells for each case. Results were described in percentage.

In the IHC staining for CHGB, CART, synaptophysin, and CHGA, the tissue microarrays technique was used (Manual Tissue Microarrayer 1—Beecher Instruments). Whenever possible, 3 tumor areas of interest were selected and marked by pathologist 1, both in HE-stained slides and in the respective donor tissue paraffin blocks, and a spreadsheet containing the corresponding block numbers was elaborated for mapping purposes. The donor block was perforated in the exact region marked by the pathologist and the material was transferred to the recipient block. After executing all the spots, the recipient block was placed in the oven at 60 °C for paraffin softening and spot leveling, treated with layers paraffin to preserve immunoreactivity, and was cut for slide preparation. To carry out antigen retrieval, the slides were immersed in a Tris-EDTA solution, pH 9.0 (K800421-2, Agilent) and steamed-treated at 100 °C for 35 minutes. After peroxidase blocking, the slides were incubated with the primary antibody (CHGB—mouse monoclonal antibody MAB8868, 1:2000 dilution, R&D Systems, RRID: AB_3096181; CART—rabbit monoclonal antibody NBP1-91749, 1:400 dilution, Cell Signaling Technology; RRID: AB_2798480) for 30 minutes at 37 °C and then for 18 to 24 hours at 4 °C. Signal amplification was performed using EnVision FLEX+ (Agilent), followed by DAB reaction (Sigma). The slides were stained with hematoxylin and covered with Entellan (Merck). Adrenal tissue slides were used as controls for CHGB and CART. The results were assessed by pathologist 2 as a percentage of positive cells (0%-100%) and in intensity (weak [1], moderate [2], and strong [3]). Using that data the IHC positivity index (PI) was calculated (percentage × intensity). The final PI value was calculated as the average of the results obtained for the available spots in each case and categorized as negative (<30), very weak (30-70), weak (71-150), moderate (151-199), and strong (≥200). Automated IHC reactions (Benchmark ULTRA Ventana) for synaptophysin (IHC-synapthophysin) and CHGA (IHC-CHGA) were used to confirm the neuroendocrine origin of the tumor. For antigen retrieval, the slides were immersed in ULTRA Conditioning (Ultra CC1, pH: 8.4, Ventana Medical Systems) for 76 minutes for synaptophysin and for 92 minutes for CHGA, and steam-treated at 95 °C. After peroxidase blocking, the slides were incubated with the primary antibodies (synaptophysin—rabbit monoclonal antibody MRQ-40, ready to use, Cell Marque, RRID:AB_3096182; and CHGA—mouse monoclonal antibody LK2H10, ready to use, Ventana, RRID:AB_2335955) for 1 hour and 36 minutes at 37 °C. Signal amplification was performed using the ultraView Universal DAB Detection Kit (Ventana Medical Systems). The slides were stained with hematoxylin and covered with Entellan (Merck). Central nervous system tissue was used as a control for synaptophysin, and gastric tissue was used as a control for CHGA. Results were assessed as positive or negative by pathologist 2.

### Definition of Metastatic and Nonmetastatic Disease

Patients were identified as having metastatic paraganglioma (MPGL) if they presented metastasis at the diagnosis of the primary tumor or during postoperative follow-up (until July 2023). Metastatic disease was suspected by recurrence of hypertension and/or other signs and symptoms of adrenergic hyperactivation and/or elevation of catecholamines or their metabolites above normal limits. It was always confirmed by imaging (CT scan and magnetic resonance imaging) and/or by nuclear medicine techniques (most frequently bone scintigraphy and ^123/131^MIBG but also OctreoScan, PET/CT-FDG and PET Gallium-68 DOTATATE PET/CT). Patients who did not show evidence of metastatic disease during the minimum follow-up period, defined based on the assessment of the maximum time interval between initial diagnosis and the detection of metastasis in patients with MPGL, were classified as having nonmetastatic PGL (NMPGL). Patients who presented with local tumor recurrence caused by tumor cell implantation during surgery (pheochromocytomatosis) [74-77] and those with inferior vena cava thrombosis without concomitant metastasis were excluded. To investigate prognostic predictors in patients with MPGL, this group was divided into 2 subgroups: aggressive MPGL (aMPGL), patients who died earlier in the period following surgery, and indolent MPGL (iMPGL), patients who survived for a prolonged period, with or without disease, at the last assessment.

### Statistical Analysis

The results were expressed as absolute values and frequency percentages for categorical variables and as mean ± SD, median, and minimum and maximum values for numerical variables. Univariate analysis was performed to test the association between each variable and metastasis as an outcome. Categorical variables were analyzed using the chi-square test with Fisher exact test or likelihood ratio tests when necessary. Data normality in the studied population was assessed using the Shapiro-Wilk test. The *t* test was used for numerical variables with normal distribution, and the Mann-Whitney test was used for numerical variables that did not follow a normal distribution. It was possible to calculate the odds ratio (OR) for variables present in both groups with a minimum number of 1. Subsequently, multivariable analysis was conducted to identify which variables were independently associated with the outcome of metastasis. Considering the total number of positive outcomes studied (metastatic tumors), we calculated that to establish good reliability for the model we should select 1 variable for every 5 outcomes for this analysis. Cases with one or more missing data in the selected variables were omitted. The choice of variables included in the multivariable analysis followed these criteria: variables with a *P* value of less than .05 in the univariate analysis (MPGL vs NMPGL); and histological variables that, according to the pathologists, did not represent the same histological phenomenon. If the same histological phenomenon was seen, the higher OR variable and/or the easier to identify or more reproducible variable was chosen. The multivariable analysis was performed by adjusted multiple logistic regression using the stepwise backward selection method. The β coefficient (β coef) generated in the multivariable analysis model was used to weight each variable by multiplying its value by 10 and rounding it up to the next whole number [78, 79]. With the numbers obtained we developed a prognostic score for PGL. The estimated probability of metastasis was calculated using the same coefficients in one equation ($P=\frac{e^{\left(\beta_{0}+\beta_{1}+\beta_{2}+\ldots +\beta_{n}\right)}}{1+e^{\left(\beta_{0}+\beta_{1}+\beta_{2}+\ldots +\beta_{n}\right)}}$ where *P* = outcome probability, *e* = Euler number, β_0_ = constant of regression equation, β_1_, β_2_ … β_n_ = regression coefficients of each variable) and with the data obtained a curve to estimate the risk of metastasis was built. Receiver operating characteristic (ROC) curves were generated to assess the performance of the predictors in differentiating cases with and without metastasis by calculating the area under the curve (AUC). Sensitivity and specificity were obtained from inflection points of the curve and positive predictive values (PPVs) and negative predictive values (NPVs) were calculated. The 95% CIs were established for each of these parameters. The cutoff point with best sensitivity and specificity together was calculated using the Youden method [80]. In the analysis of disease-specific survival (DSS), only deaths related to disease (PGL) were considered. Kaplan-Meier curves were generated for both groups (MPGL and NMPGL). The same characteristics used for determining metastatic potential in addition to time of onset and site of metastasis were analyzed in the 2 subgroups of MPGL (aMPGL and iMPGL) to assess prognostic predictors in MPGL. Kaplan-Meier curves were generated for patients with MPGL considering characteristics associated with worse prognosis. Survival curves were compared using the log-rank test.

In all the analyses carried out, *P* values less than .05 were considered statistically significant. Data analysis was performed using IBM SPSS Statistics for Windows from IBM Corp. version 27.0 released in 2020 by IBM Corp.

## Results

During the period of analysis, 263 patients were identified with a diagnosis of PGL. Out of the 263 patients identified, 13.3% (35/263) were diagnosed with MPGL. The diagnosis of metastasis was synchronous with primary tumor diagnosis in 57.1% (20/35) and metachronous in 42.9% (15/35) of the cases. In patients with metachronous metastases, the disease-free interval ranged from 12 to 84 months after primary tumor surgery, with a mean of 44 months. The main sites of metastasis were bones (68.6%), lymph nodes (57.1%), lungs (31.4%), and liver (28.6%) (Table 1).

**Table 1.** Time of detection and site of metastasis in metastatic paraganglioma

Variable	MPGL (n = 35)
Synchronous metastasis*^a^*	57.1% (20/35)
Metachronous metastasis*^b^*	42.9% (15/35)
Time to metachronous metastasis detection, mo	44.3 ± 57 36 (12-84)
Metastasis only to lymph nodes*^c^*	22.9% (8/35)
Metastasis to lymph nodes	57.1% (20/35)
Metastasis to bones	68.6% (24/35)
Metastasis to liver	28.6% (10/35)
Metastasis to lung	31.4% (11/35)

Results expressed as mean ± SD, median (minimum value-maximum value), or percentage (n positive/n available).

^*a*^Synchronous metastasis: less than 12 months after primary tumor diagnosis.

^*b*^Metachronous metastasis: 12 months or more after primary tumor diagnosis.

^*c*^Patients with metastasis only to regional lymph nodes.

**Figure 1.**
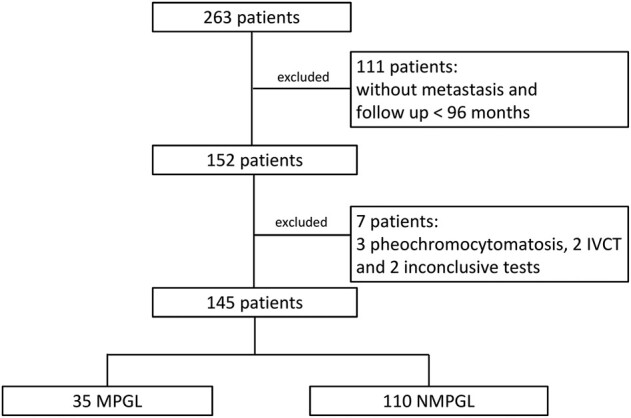
Selection of patients. IVCT, inferior vena cava thrombosis; MPGL, metastatic paraganglioma; NMPGL, nonmetastatic paraganglioma.

The maximum period in which metastatic disease was observed in patients with MPGL was 84 months after removal of the primary tumor. Therefore, patients who were free of metastatic disease with follow-up of 96 months or more after primary tumor surgery were considered as having NMPGL. Out of the initially studied 263 patients, 111 patients were excluded because of insufficient follow-up, even though they did not present with metastasis. Additionally, 7 other patients were excluded: 2 patients due to an isolated inferior vena cava thrombosis at the time of primary tumor surgery, 3 patients who developed pheochromocytomatosis during follow-up after surgery, and 2 patients who had inconclusive findings in laboratory tests. Therefore, with a final selection of 145 patients, data from the MPGL group (35 patients) and the NMPGL group (110 patients) were analyzed and compared (Fig. 1).

### Clinical Data

The comparison of patient clinical, genetic, and progression characteristics is shown in Table 2. There was no difference in terms of age and sex of the patients. Clinical diagnosis predominated in both groups and was more frequent in patients with metastatic disease (*P* = .036; OR = 3.2), whereas diagnosis by genetic screening only occurred in NMPGL (*P* = .012). Genetic investigation was performed in 106 of 145 (73%) of the patients, 21 from the MPGL group and 85 from the NMPGL group, and was more frequently positive in the NMPGL group (*P* = .011). In only 6 patients the genetic diagnosis was based on clinical presentation of MEN 2 (PGLAd + medullary thyroid carcinoma + primary hyperparathyroidism). The presence of PV in the *RET* and *VHL* genes occurred only in patients with NMPGL with *P* = .001 for *RET*; PV in the *SDHB* and *NF1* genes were present in both groups, with *P* = .014 and OR = 6 for *SDHB*; PV in the *TMEM127*, *SDHD*, and *FH* genes were present only in patients in the NMPGL group, whereas PV in the *SDHA* gene occurred only in MPGL. Follow-up was shorter in MPGL vs NMPGL (median 144 months vs 168 months; *P* = .033) (see Table 2).

**Table 2.** Clinical and genetic data of metastatic paraganglioma vs nonmetastatic paraganglioma

Variable	MPGL (n = 35)	NMPGL (n = 110)	*P*	OR	95% CI
Age, y	36.4 ± 17.0 35 (10-70)	40.4 ± 16.6 38 (4-78)	.212	0.985	0.962-1.009
Female	51.4% (18/35)	64.5% (71/110)	.165	0.58	0.27-1.26
Clinical presentation					
Symptoms	88.2% (30/34)	70.1% (68/97)	.036*^a^*	3.2	1.03-9.90
Incidentalomas	11.8% (4/34)	13.4% (13/97)	>.999	0.86	0.26-2.85
Genetic screening	0% (0/34)	16.5% (16/97)	.012*^a^*	NE	NE
Positive genetic diagnosis	38.1% (8/21)	68.2% (58/85)	.011*^a^*	0.29	0.11-0.77
Gene					
*RET*	0% (0/21)	36.4% (31/85)	.001*^a^*	NE	NE
*VHL*	0% (0/21)	15.3% (13/85)	.067	NE	NE
*SDHB*	23.8% (5/21)	4.7% (4/85)	.014*^a^*	6.33	1.53-26.18
*NF1*	9.5% (2/21)	2.4% (2/85)	.175	4.37	0.58-33.01
*TMEM127*	0% (0/21)	7.1% (6/85)	.596	NE	NE
*SDHD*	0% (0/21)	1.2% (1/85)	>.999	NE	NE
*SDHA*	4.8% (1/21)	0% (0/85)	.198	NE	NE
*FH*	0% (0/21)	1.2% (1/85)	>.999	NE	NE
Negative	61.9% (13/21)	31.7% (27/85)			
Follow-up, mo	155.9 ± 111.0 144 (12-384)	188.7 ± 81 168 (96-504)	.033	0.995	0.99-1

Results expressed as mean ± SD, median (minimum-maximum), or percentage (n positive/n available).

Abbreviations: FH, fumarate hydratase; MPGL, metastatic paraganglioma group; NE, not possible to estimate; NF1, neurofibromatosis type 1; NMPGL, nonmetastatic paraganglioma group; OR, odds ratio; RET, rearranged during transfection; SDHA, succinate dehydrogenase subunit A; SDHB, succinate dehydrogenase subunit B; SDHD, succinate dehydrogenase subunit D; TMEM127, transmembrane protein 127; VHL, von Hippel-Lindau.

^*a*^Significant *P* less than .05.

Biochemical diagnosis and tumor functionality data are shown in Table 3. Catecholamines and related metabolites data were similar in both patient groups, except for 24-hour urinary vanilmandelic acid (*P* = .021), 24-hour urinary noradrenaline (*P* = .048), 24-hour urinary dopamine (*P* = .017), and plasmatic noradrenaline (*P* = .027), which were higher in MPGL; however, with borderline OR for 24-hour urinary vanilmandelic acid (OR = 1.03) and nonsignificant for plasmatic noradrenaline (OR = 1; CI, 1.00-1.00) and 24-hour urinary dopamine (OR = 4.396; CI, 0.482-40.104). The concentrations of 24-hour urinary noradrenaline were higher in MPGL vs NMPGL (median 350 mcg/24 hours vs 81 mcg/24 hours, OR = 2.153). There was no difference between the groups in terms of functionality (functional or nonfunctional) or in the type of functionality (adrenergic, noradrenergic, or nonfunctional) (see Table 3).

**Table 3.** Production of catecholamines and functional type of metastatic paraganglioma vs nonmetastatic paraganglioma

Variable (reference range)	MPGL (n = 35)	NMPGL (n = 110)	*P*	OR	95% CI
VMAU (≤12 mg/24 h)	47.0 ± 30.6 35.2 (15-96.8) (11/35)	27.2 ± 23.8 19.2 (1.64-96.8) (51/110)	.021*^a^*	1.03	1.01-1.05
tMnU (0.05-1.2 mcg/mg Cr)	5.4 ± 5.4 3.4 (0.14-20) (21/35)	4.0 ± 5.5 1.8 (0.1-36.9) (78/110)	.144	1.04	0.96-1.13
AU (0.5-20 mcg/24 h)	116 ± 229.6 22.5 (0-899) (18/35)	113.0 ± 274.5 15 (0-1277) (58/110)	.495	1.04	0.14-7.75
NAU (14-80 mcg/24 h)	1237.7 ± 1687.1 350 (9-5187) (18/35)	372.0 ± 577.0 81 (7-3066) (60/110)	.048*^a^*	2.153	1.21-3.82
DopaU (65-400 mcg/24 h)	1675.7 ± 5547.2 361.5 (9-23 888) (18/35)	285.0 ± 229.5 250 (0-1296) (59/110)	.017	4.396	0.482-40.104
AP (0-75 pg/mL)	461.3 ± 794.1 40.2 (0-2770) (15/35)	367.5 ± 713.1 27.5 (0-3645) (58/110)	.551	1.18	0.55-2.54
NAP (40-268 pg/mL)	11 435.4 ± 15 439.5 3422 (180-50 421) (14/35)	2739.1 ± 4017.8 1008 (0-17 437) (59/110)	.027*^a^*	1.000	1.000-1.000
DopaP (0-83 pg/mL)	46.0 ± 157.8 0 (0-593) (14/35)	109.2 ± 653.8 0 (0-4645) (51/110)	.814	0.759	0.16-3.69
MnP (<0.5 nmol/L)	2.7 ± 2.6 2.8 (0.2-5.9) (5/35)	0.7 ± 0.6 0.5 (0-1.6) (9/110)	.252	2.41	0.82-7.05
NMnP (<0.9 nmol/L)	20.2 ± 22.0 12.6 (0.7-57.9) (5/35)	3.9 ± 6.1 0.9 (0.5-16.6) (9/110)	.072	1.17	0.96-1.42
Functional/Nonfunctional tumor Functional Nonfunctional	90% (27/30) 10% (3/30)	89% (81/91) 11% (10/91)	>.999	1.11	0.29-4.34
Adrenergic	54.5% (12/22)	55.6% (35/63)	.935	0.96	0.36-2.55
Noradrenergic	31.8% (7/22)	28.6% (18/63)	.774	1.17	0.41-3.34
Nonfunctional	13.6% (3/22)	15.9% (10/63)	>.999	0.84	0.21-3.37

Results expressed as mean ± SD, median (minimum-maximum), or percentage (n positive/n available).

Abbreviations: AP, plasmatic adrenaline; AU, 24-hour urinary adrenaline; DopaP, plasmatic dopamine; DopaU, 24-hour urinary dopamine; MnP, plasmatic metanephrine; MPGL, metastatic paraganglioma group; NAU, 24-hour urinary noradrenaline; NMnP, plasmatic normetanephrine; NMPGL, nonmetastatic paraganglioma group; NP, plasmatic noradrenaline; OR, odds ratio; tMnU, 24-hour urinary total metanephrines; VMAU, 24-hour urinary vanilmandelic acid.

^*a*^Significant *P* less than .05.

Data regarding tumor size and location are shown in Table 4. Extra-adrenal location was more frequent in MPGL (*P* < .001; OR = 6.75). The PGLexAd were most often located in the abdomen (80.8%) and, less frequently, in the pelvis (11.5%) and head and neck (7.7%). The tumors were larger in MPGL vs NMPGL (median 7.9 cm vs 4.5 cm; *P* < .001; OR = 1.34) (see Table 4). The area under the ROC curve (AUC-ROC) for tumor size was 0.760, and tumor size with the best sensitivity and specificity to differentiate metastatic tumors was 8.1 cm [81]. When we consider this value, we observe an increased association with metastatic disease (OR = 7.5) (see Table 4).

**Table 4.** Site and size of metastatic paraganglioma vs nonmetastatic paraganglioma

Variable	MPGL (n = 35)	NMPGL (n = 110)	*P*	OR	95% CI
Tumor site					
PGLAd	57.1% (20/35)	90% (99/110)	<.001*^a^*	0.15	0.06-0.37
PGLexAd	42.9% (15/35)	10% (11/110)	<.001*^a^*	6.75	2.7-16.84
Tumor size, cm	8.3 ± 4.2 7.9 (3-20)	4.9 ± 2.8 4.5 (0.8-16)	<.001*^a^*	1.34	1.17-1.54
Tumor size ≥ 8.1 cm	50% (17/34)	11.8% (12/102)	<.001*^a^*	7.50	3.04-18.50

Results expressed as mean ± SD, median (minimum-maximum), or percentage (n positive/n available).

Abbreviations: PGLAd, paraganglioma in the adrenal medulla; MPGL, metastatic paraganglioma group; NMPGL, nonmetastatic paraganglioma group; OR, odds ratio; PGLexAd, paraganglioma in extra-adrenal location.

^*a*^Significant *P* less than .05.

### Histology

**Figure 2.**
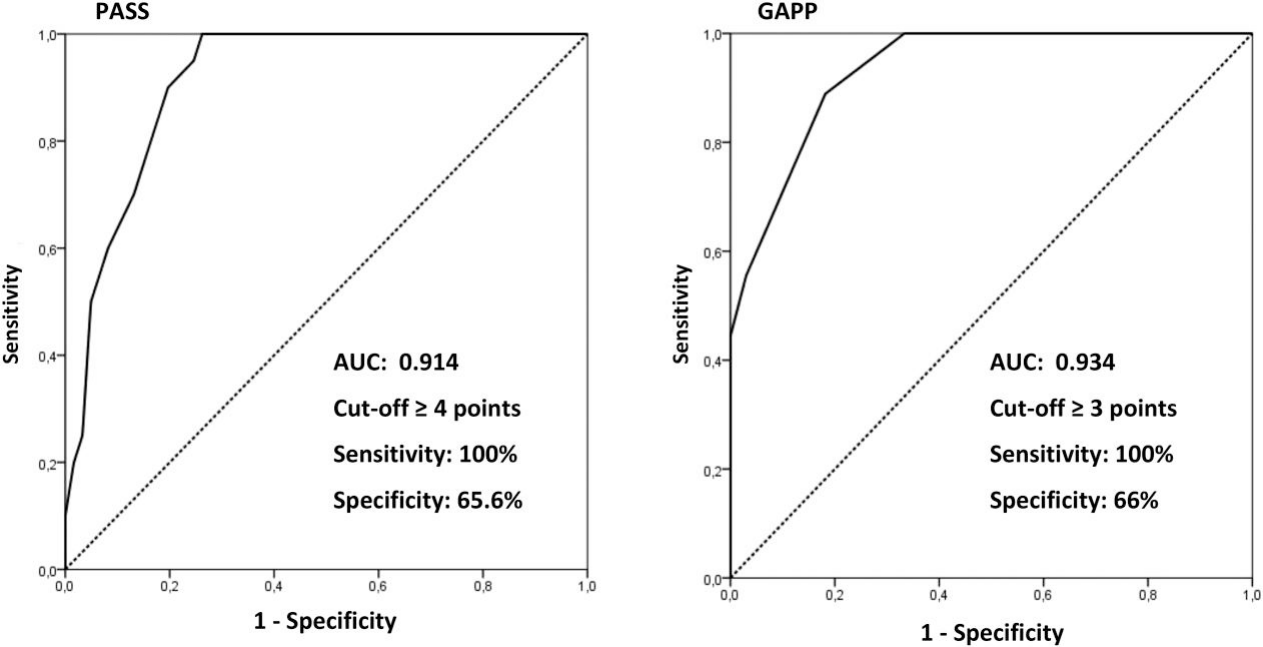
Receiver operating characteristic (ROC) curve (status variable: metastasis) to PASS and GAPP. AUC, area under the curve; GAPP, Grading system for adrenal Phaeochromocytoma and Paraganglioma; PASS, Pheochromocytoma of the Adrenal Gland Scaled Score [32, 33].

The total number of points in the PASS and GAPP scores and the frequency of histological parameters comprising them, assessed in MPGL and NMPGL, are shown in Table 5. The total points in PASS were greater in MPGL compared with NMPGL (median 9.5 points vs 2 points; *P* < .001; OR = 1.7). All the MPGL patients had a score of 4 or more points. Most histological variables were more prevalent in MPGL patients, except the presence of spindle cells and nuclear hyperchromasia (see Table 5). The AUC-ROC for the PASS score (Fig. 2) was 0.914, and the cutoff of 4 or greater showed 100% sensitivity (CI, 62.3%-77.3%), 65.6% specificity (CI, 62.3%-77.3%), 48.8% PPV (CI, 32.9%-64.9%), and 100% NPV (CI, 91.2%-100%) for detecting potentially metastatic disease.

**Table 5.** Pheochromocytoma of the Adrenal Gland Scaled Score and Grading System for Adrenal Pheochromocytoma and Paraganglioma scores in the metastatic paraganglioma group vs the nonmetastatic paraganglioma group

Variable	MPGL (n = 35)	NMPGL (n = 110)	*P*	OR	95% CI
PASS (points)*^a^*	9.5 ± 2.95 9.5 (5-17)	3.0 ± 3.33 2 (0-12)	<.001*^c^*	1.72	1.34-2.22
PASS ≥ 4 points	100% (20/20)	34.4% (21/61)	<.001*^c^*	NE	NE
Large nests or diffuse growth	95% (19/20)	29.5% (18/61)	<.001*^c^*	45.39	5.64-365.06
Central or confluent necrosis	55% (11/20)	3.3% (2/61)	<.001*^c^*	36.06	6.84-189.99
High cellularity	70% (14/20)	23% (14/61)	<.001*^c^*	7.83	2.54-24.18
Cellular monotony	35% (7/20)	11.5% (7/61)	.035*^c^*	4.15	1.24-13.93
Tumor cell spindling	25% (5/20)	11.9% (7/59)	.168	2.48	0.69-8.94
Mitotic figures > 3/10 HPF	40% (8/20)	3.3% (2/60)	<.001*^c^*	19.33	3.64-102.65
Atypical mitotic figures	25% (5/20)	1.6% (1/61)	.003*^c^*	20	2.17-184.21
Extension into adipose tissue	36.8% (7/19)	11.7% (7/60)	.033*^c^*	4.42	1.30-14.97
Vascular invasion	52.6% (10/19)	26.2% (16/61)	.032*^c^*	3.13	1.08-9.08
Capsular invasion	55% (11/20)	29.5% (18/61)	.039*^c^*	2.92	1.03-8.25
Profound nuclear pleomorphism	55% (11/20)	21.7% (13/60)	.005*^c^*	4.42	1.51-12.93
Nuclear hyperchromasia	30% (6/20)	27.9% (17/61)	.854	1.11	0.37-3.36
GAPP (points)*^b^*	5.1 ± 1.5 5 (3-7)	1.7 ± 1.60 1 (0-9)	<.001*^c^*	4.68	1.45-15.06
GAPP ≥ 3 points	100% (15/15)	34% (17/50)	<.001*^c^*	NE	NE
GAPP					
WD	0% (0/15)	66% (33/50)	<.001*^c^*	NE	NE
MD	80% (12/15)	32% (16/50)	<.001*^c^*	8.5	2.10-34.39
PD	20% (3/15)	2% (1/50)	.036*^c^*	12.25	1.1-128.39

Results expressed as mean ± SD, median (minimum-maximum), or percentage (n positive/n available).

Abbreviations: GAPP, Grading System for Adrenal Pheochromocytoma and Paraganglioma [33]; HPF, high-power field; MD, moderately differentiated; MPGL, metastatic paraganglioma group; NE, not possible to estimate; NMPGL, nonmetastatic paraganglioma group; OR, odds ratio; PASS, Pheochromocytoma of the Adrenal Gland Scaled Score [32]; PD, poorly differentiated; WD, well differentiated.

^*a*^PASS histologic parameters were assessed in 19 to 20 MPGL and in 59 to 61 NMPGL patients.

^*b*^GAPP score was assessed in 15 MPGL and in 50 NMPGL patients.

^*c*^Significant *P* less than .05.

The number of total points in the GAPP score were also greater in MPGL vs NMPGL (median 5 points vs 1 point; *P* < .001; OR = 4.68). All the patients with MPGL had tumors classified as moderately or poorly differentiated, while patients with NMPGL had well- or moderately differentiated tumors, and rarely poorly differentiated tumors (see Table 5). The AUC-ROC for the GAPP score (see Fig. 2) was 0.934, and the cutoff of 3 or greater showed 100% sensitivity (CI, 78.2%-100%), 66% specificity (CI, 51.2%-78.8%), 46.9% PPV (CI, 29.1%-65.3%), and 100% NPV (CI, 89.4%-100%) for detecting potentially metastatic disease.

### Immunohistochemistry

All the tumors were positive for IHC-CHGA and IHC-synaptophysin, except for one PGLM that was negative for CHGA but positive for synaptophysin, confirming the neuroendocrine origin of the tumors studied.

The IHC assessment of the Ki-67, CHGB, and CART markers is shown in Table 6. The Ki-67 IHC indices were higher in the MPGL group vs NMPGL (median 2.3% vs 0.4%; *P* = .001; OR = 1.467). The percentage of MPGL with IHC-Ki-67 of 3% or greater was higher than in NMPGL (45.5% vs 11.9%; *P* = .002; OR = 6.19). The AUC-ROC for IHC-Ki-67 was 0.773 and the cutoff of 3% or greater showed 46% sensitivity but 88% specificity for determining metastatic potential [81]. The PI-CHGB was lower in MPGL vs NMPGL (median 70 vs 166.7; *P* = .023; OR = 0.994). The AUC-ROC for this parameter was 0.671 and the value of 200 or greater showed 85% sensitivity and 45.6% specificity for predicting nonmetastatic disease [81]. The PI-CART was similar in both tumor groups (median 10 in both groups; *P* = .906) (see Table 6).

**Table 6.** Immunohistochemistry for Ki-67, cocaine- and amphetamine-regulated transcript, and chromogranin B in the metastatic paraganglioma group vs the nonmetastatic paraganglioma group

Variable	MPGL (n = 35)	NMPGL (n = 110)	*P*	OR	95% CI
Ki-67 (%)*^a^*	3.2 ± 3.1 2.3 (0.1-9.5)	1.0 ± 1.7 0.4 (0.1-8)	.001*^d^*	1.467	1.164-1.846
Ki-67 ≥ 3%	45.5% (10/22)	11.9% (7/59)	.002*^d^*	6.19	1.96-19.59
Ki-67 > 5%	22.7% (5/22)	5.1% (3/59)	.031*^d^*	5.49	1.19-25.38
PI-CHGB*^b^*	99.67 ± 81.03 70 (0-250)	165.0 ± 111.8 166.7 (6.7-300)	.023*^d^*	0.994	0.99-0.02
PI-CHGB ≥ 200	15% (3/20)	45.6% (26/57)	.015*^d^*	0.21	0.06-0.80
PI-CART*^c^*	31.60 ± 46.05 10 (0-186.7)	24.8 ± 36.2 10 (0-170)	.906	1.01	0.99-1.02

Results expressed as mean ± SD, median (minimum-maximum), or percentage (n positive/n available).

Abbreviations: CART, cocaine and amphetamine regulated transcript; CHGB, chromogranin B; IHC, immunohistochemistry; MPGL, metastatic paraganglioma group; NMPGL, nonmetastatic paraganglioma group; OR, odds ratio; PI, positivity index of immunohistochemistry.

^*a*^IHC-Ki-67 was assessed in 22 MPGL and in 59 NMPGL patients.

^*b*^IHC-CHGB was assessed in 20 MPGL and in 57 NMPGL patients.

^*c*^IHC-CART was assessed in 20 MPGL and in 57 NMPGL patients.

^*d*^Significant *P* less than .05.

### Prognostic Score of Paragangliomas

The PSPGL was developed based on the results of the multivariable analysis. For this analysis, we chose 7 variables (1 variable for every 5 outcomes). Initially, we selected those variables that had *P* less than .05 in the univariate analysis, thus, we would have 10 histological variables (diffuse growth and/or large nests, central or confluent necrosis, high cellularity, cellular monotony, > 3 mitoses/10 HPF, atypical mitotic figures, extension into adipose tissue, vascular invasion, capsular invasion, and profound nuclear pleomorphism) and 6 nonhistological variables (adrenergic symptoms, extra-adrenal tumor location, PV in the *SDHB* gene, concentrations of 24-hour urinary vanilmandelic acid and 24-hour urinary noradrenaline, and tumor size ≥8.1 cm). Among the 10 initially selected histological variables, some represented the same histological phenomenon: 1—diffuse growth and/or large nests and central or confluent necrosis (a central or confluent necrosis occurs in the center of a large nest or extends diffusely through several large nests), we opted for the variable necrosis, as it is more reproducible; 2—cellular monotony and profound nuclear pleomorphism (cells exhibiting a monotonous pattern generally have deep nuclear pleomorphism, with a high nucleus-cytoplasm index) [32], we chose the variable cellular monotony because of its higher interobserver agreement [37]; 3— more than 3 mitoses/10 HPF and atypical mitotic figures (atypical mitotic figures are more common with a higher mitotic index), we opted for more than 3 mitoses/10 HPF due to its higher interobserver agreement [37]. 4—vascular invasion, capsular invasion, and extension into adipose tissue (all represent local tumor invasiveness), we chose the variable extension into adipose tissue because this variable received greater weight in the PASS score and had a higher OR in the univariate analysis. High cellularity appears to represent an isolated phenomenon and was also selected (total histological variables = 5). Ki-67 was not selected because it reflects the mitotic index, already included among the histological variables. Considering the 6 nonhistological variables already chosen, we would have a total of 11 variables; among them we chose the 7 variables with the highest OR in the univariate analysis. The nonhistological variables were tumor size 8.1 cm or larger (OR = 7.5), extra-adrenal tumor location (OR = 6.75), and PV in the *SDHB* gene (OR = 6.33), and the histological variables were necrosis (OR = 36.6), more than 3 mitoses/10 HPF (OR = 19.33), high cellularity (OR = 7.83), and extension into adipose tissue (OR = 4.42). Multivariable analysis showed that only 4 variables remained independently related to the occurrence of metastasis: presence of central or confluent necrosis, more than 3 mitoses/10 HPF, extension into adipose tissue, and extra-adrenal location (Table 7).

**Table 7.** Predictors of metastatic disease in the metastatic paraganglioma group vs the nonmetastatic paraganglioma group

Variables	MPGL	NMPGL	*P*	OR (95% CI)	β coef
Central or confluent necrosis	55% (11/20)	3.3% (2/61)	<.001*^a^*	507.01 (16.39-15 688.6)	6.229
Mitotic figures > 3/10 HPF	40% (8/20)	3.3% (2/60)	.004*^a^*	185.99 (5.26-6580.84)	5.226
Extension into adipose tissue	36.8% (7/19)	11.7% (7/60)	.011*^a^*	39.01 (2.34-650.10)	3.664
Extra-adrenal location	42.9% (15/35)	10% (11/110)	.009*^a^*	37.26 (2.49-556.78)	3.618
*SDHB*	23.8% (5/21)	4.7% (4/85)	NS	—	—
Tumor size ≥ 8.1 cm	50% (17/34)	11.8% (12/102)	NS	—	—
High cellularity	70% (14/20)	23% (14/61)	NS	—	—

Results expressed as mean ± SD, median (minimum-maximum) or percentage (n positive/n available).

Abbreviations: β coef, β coefficient; HPF, high-power field; MPGL, metastatic paraganglioma group; NMPGL, nonmetastatic paraganglioma group; NS, nonsignificant; OR, odds ratio.

^*a*^Significant *P* less than .05.

**Figure 3.**
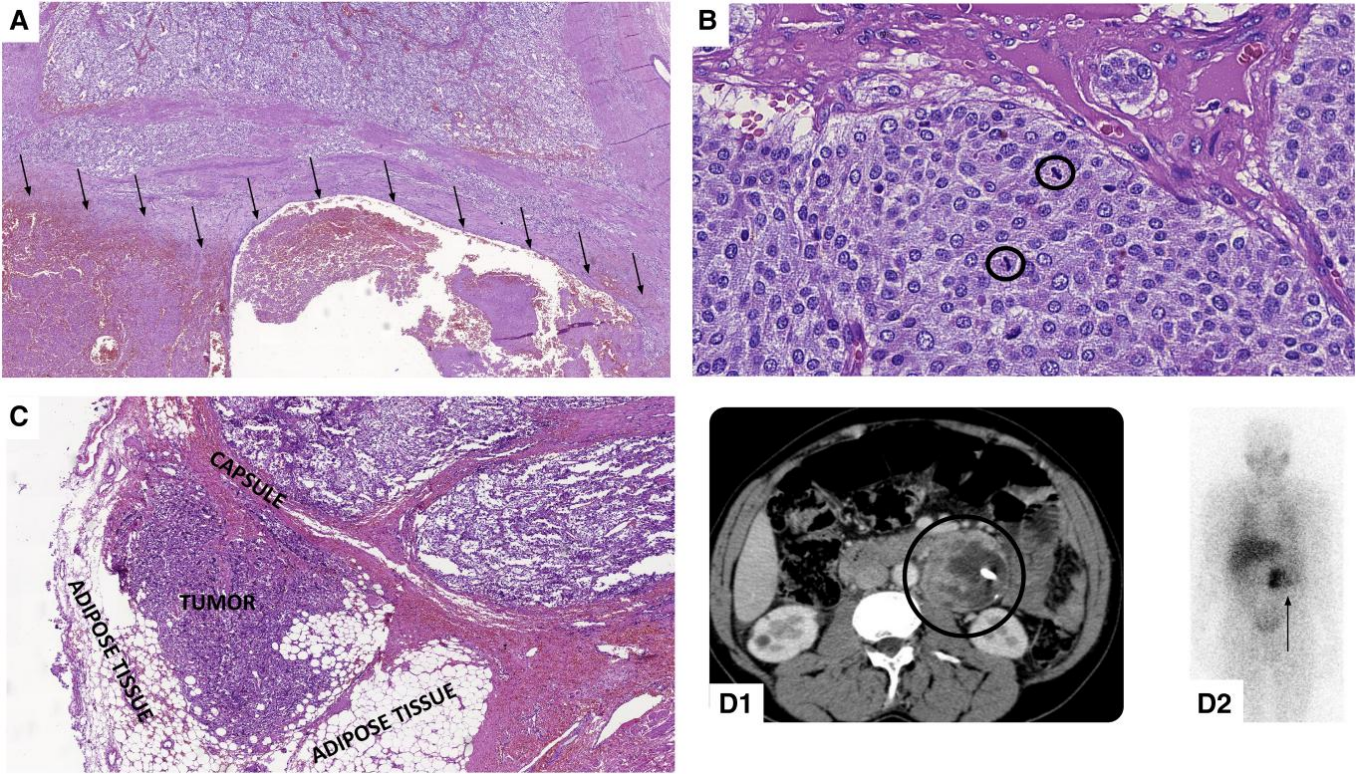
Predictors of metastatic disease of prognostic score in paragangliomas (PSPGL). A, Tumor with extensive area of necrosis (underside—arrows). B, Tumor with high mitotic index (2 mitosis/1 HPF). Circle: mitosis. C, Tumor with extension into adipose tissue. D, Extra-adrenal paraaortic paraganglioma (left) seen on computed tomography (circle) (D1) and in metaiodobenzylguanidine scintigraphy (MIBG) (arrow) (D2).

Each variable received a value equal to its β coef multiplied by 10, rounded up to the nearest whole number [78, 79]. The sum of these values was 187, and the percentage participation of each variable in decreasing order was 33% for necrosis, 28% for more than 3 mitoses/HPF, 20% for extension into adipose tissue, and 19% for extra-adrenal location. The weight assigned to each variable was equal in absolute value to the percentage, and with these values we developed the PSPGL, which ranges from 0 to 100 points (Table 8). Fig. 3 illustrates these parameters observed in the patients studied.

**Table 8.** Prognostic Score in Paragangliomas

Variable	β coefficient × 10 (%)	PSPGL
Central or confluent necrosis	62 (33%)	33
Mitotic figures > 3/10 HPF	52 (28%)	28
Extension into adipose tissue	37 (20%)	20
Extra-adrenal location	36 (19%)	19
Total score	187 (100%)	100

Abbreviations: HPF, high-power field; PSPGL, Prognostic Score in Paragangliomas.

**Figure 4.**
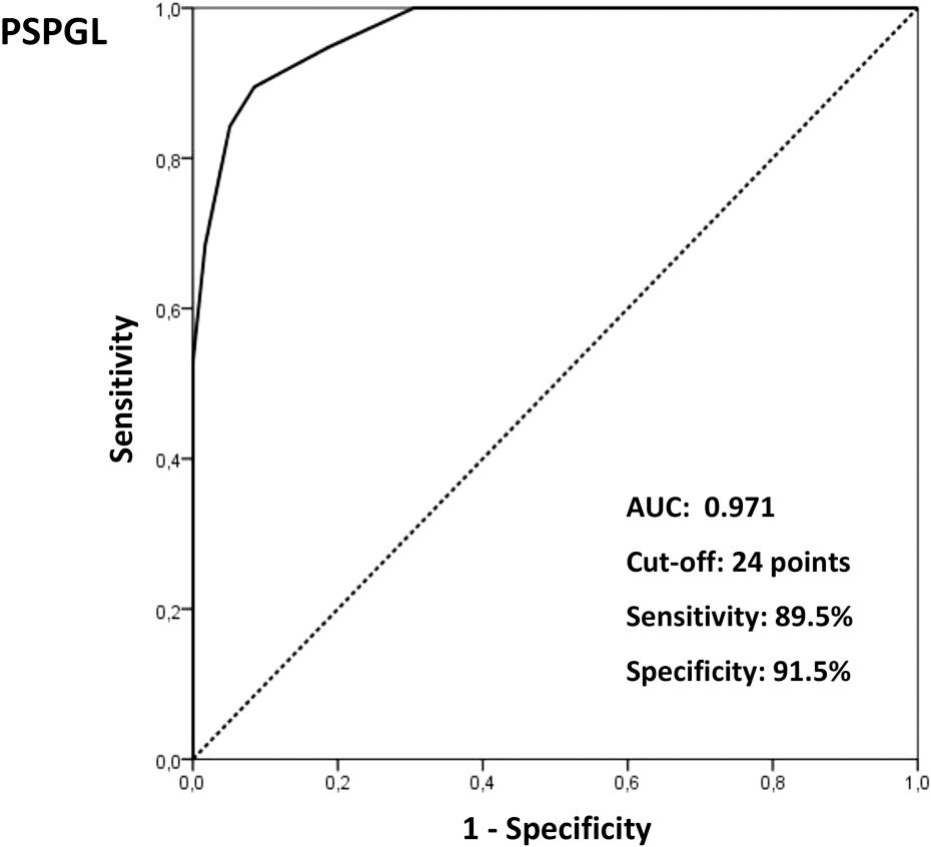
Receiver operating characteristic (ROC) curve (status variable: metastasis) to PSPGL. AUC, area under the curve; PSPGL, Prognostic Score of Paragangliomas.

The AUC-ROC for the PSPGL (Fig. 4) was 0.970, and a cutoff of 24 showed 89.5% sensitivity (CI, 66.9%-98.7%), 91.5% specificity (CI, 81.3%-97.2%), 77.3% PPV (CI, 54.6%-92.2%), and 96.4% NPV (CI, 87.7%-99.6%) in identifying metastatic potential. Table 9 shows these parameters (sensitivity, specificity, PPV, and NPV) of PASS, GAPP, and PSPGL and their respective 95% CIs. The comparison of CIs showed similar sensitivity, PPV, and NPV among the 3 scores and higher specificity for the PSPGL (see Table 9).

**Table 9.** Sensitivity, specificity, positive predictive value, and negative predictive value of the Pheochromocytoma of the Adrenal Gland Scaled Score, Grading System for Adrenal Pheochromocytoma and Paraganglioma, and Prognostic Score in Paragangliomas

Score	Sensitivity% (95% CI)	Specificity% (95% CI)	PPV% (95% CI)	NPV% (95% CI)
PASS ≥ 4	100 (83.2-100)	65.6 (52.3-77.3)	48.8 (32.9-64.9)	100 (91.2-100)
GAPP ≥ 3	100 (78.2-100)	66 (51.2-78.8)	46.9 (29.1-65.3)	100 (89.4-100)
PSPGL ≥ 24	89.5 (66.9-98.7)	91.5 (81.3-97.2)	77.3 (54.6-92.2)	96.4 (87.7-99.6)

Results obtained in the studied population.

Abbreviations: GAPP, Grading System for Adrenal Phaeochromocytoma and Paraganglioma [33]; NPV, negative predictive value; PASS, Pheochromocytoma of the Adrenal Gland Scaled Score [32]; PPV, positive predictive value; PSPGL, Prognostic Score in Paragangliomas.

We calculated the PSPGL for tumors with information on these 4 characteristics and compared the data obtained in the MPGL (19 tumors) vs NMPGL (59 tumors) groups. The total points in the PSPGL were higher in MPGL vs NMPGL (median 19 points [19-81] vs 0 points [0-39]; *P* < .001; OR = 1.98). A score of 24 or more was achieved in 89.5% of MPGL vs 8.5% of NMPGL (*P* < .001; OR = 91.8) (Table 10).

**Table 10.** Prognostic Score in Paragangliomas in the metastatic paraganglioma group vs the nonmetastatic paraganglioma group

Variable	MPGL (19/35)*^a^*	NMPGL (59/110)*^a^*	*P*	OR	95% CI
PSPGL	46.3 ± 17.6 19 (19-81)	7.0 ± 11.2 0 (0-39)	<.001*^b^*	1.98	1.43-2.74
PSPGL ≥ 24	89.5% (17/19)	8.5% (5/59)	<.001*^b^*	91.8	16.31-516.8

Results expressed as mean ± SD, median (minimum-maximum), or percentage (n positive/n available).

Abbreviations: MPGL, metastatic paraganglioma group; NMPGL, nonmetastatic paraganglioma group; OR, odds ratio; PSPGL, Prognostic Score in Paragangliomas.

^*a*^PSPGL was calculated only for tumors with available data for the 4 parameters included in the score.

^*b*^Significant *P* less than .05.

**Figure 5.**
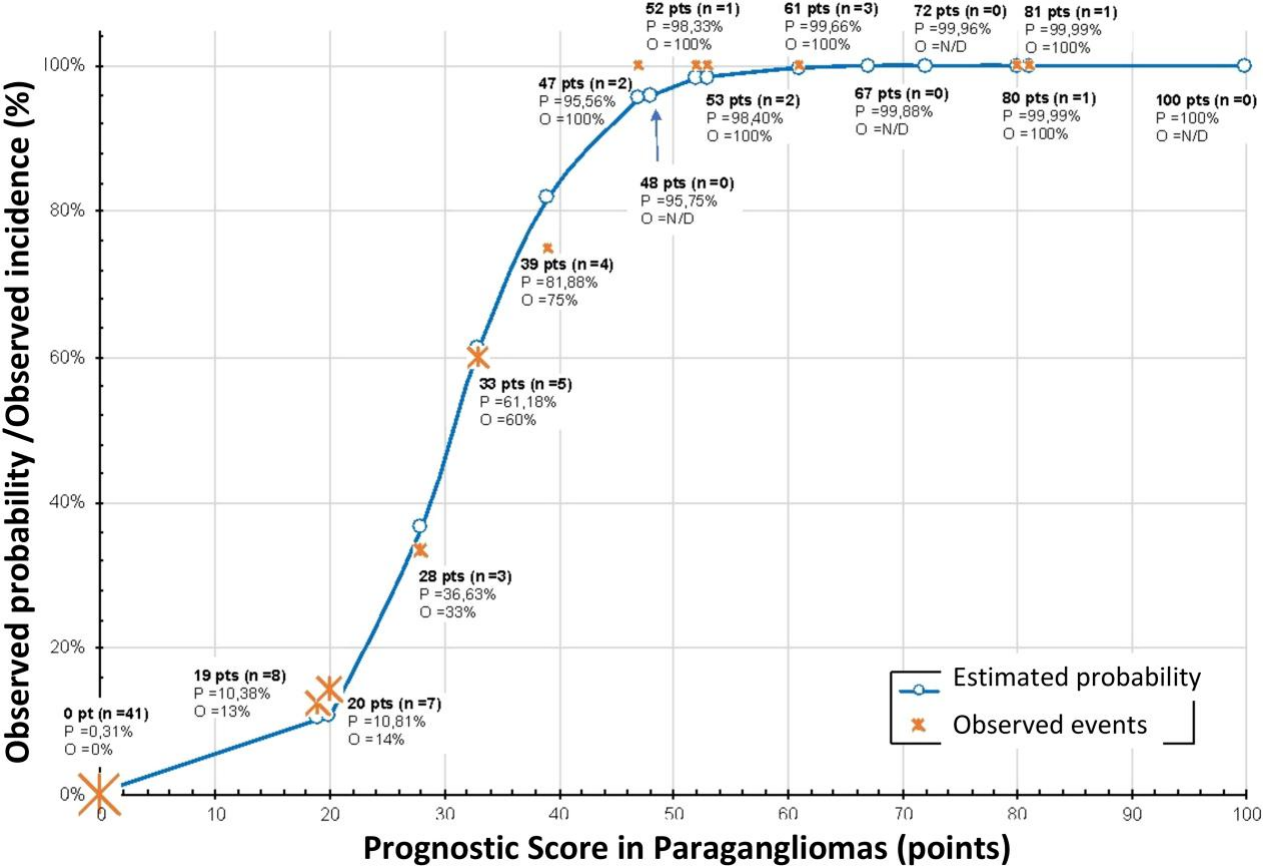
Estimated probability vs observed incidence related to PSPGL. The incidence indicators at each point represent proportional size to number of observed patients in each situation. N/D, not detected; O, observed incidence; P, estimated probability; Pts, points.

We generated a curve based on the coefficients of the logistic regression and correlated the PSPGL score with the probability of metastasis (Fig. 5). On observing the curve, we can conclude that the chance of metastatic disease is high (∼80%-100%) in patients with tumors with PSPGL of 40 or more, intermediate for PSPGL greater than 20, and less than or equal to 39, low (∼10%) in patients with tumors with a score of 20 or less, and practically null in patients with a score of zero. On the same curve, we indicated the actual occurrence of metastases and found that the estimated probability is very close to or equal to the observed incidence of metastases (see Fig. 5).

### Prognostic Factors in Paragangliomas

**Figure 6.**
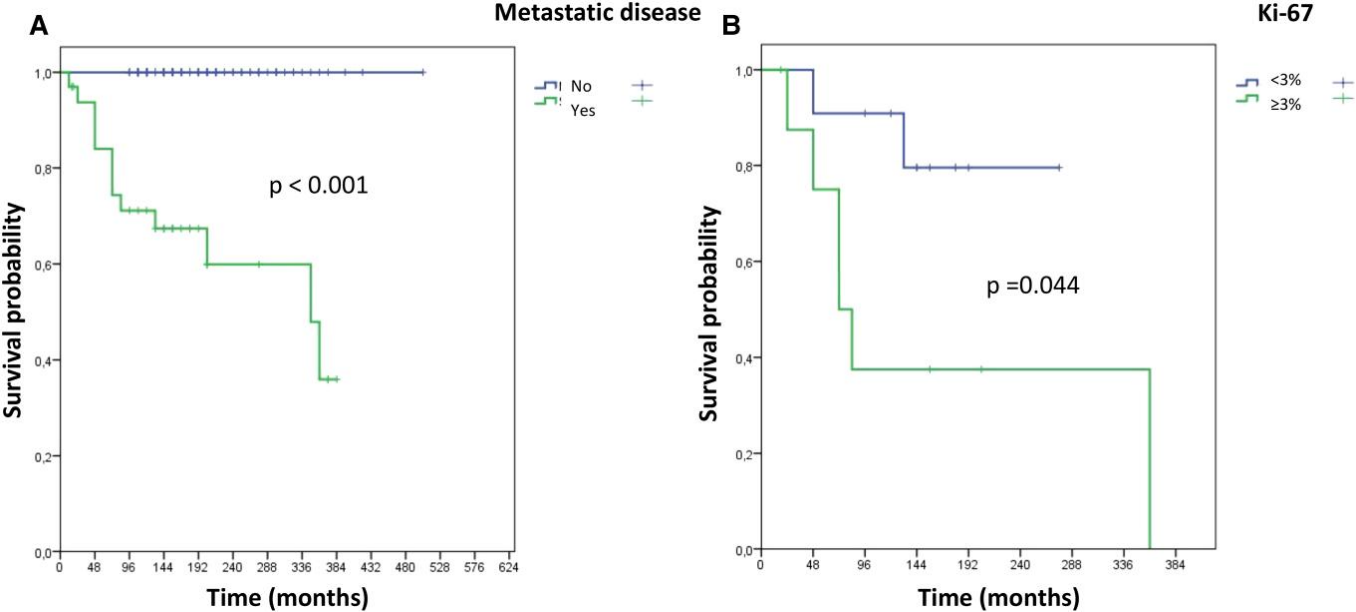
A, Disease-specific survival for patients with metastatic paraganglioma (MPGL) and nonmetastatic PGL. B, Disease-specific survival for patients with MPGL: immunohistochemistry–Ki-67 less than 3% or greater than or equal to 3%. Significant *P* less than .05.

We performed the analysis of DSS using the Kaplan-Meier method. Follow-up for patients with NMPGL was a median of 168 months (94-504 months) and no deaths related to the diagnosis of the disease were observed, resulting in a DSS of 100%. In patients with MPGL, the median was 144 months (12-384 months) and there was great variability in the clinical course of the disease (Fig. 6A). In this group, 3 patients were lost to follow-up after surgery, and out of the remaining 32, 13 patients died. The deaths occurred within 72 months or less in 8 patients (median = 48 months [12-72 months]) and they were defined as aMPGL, while 4 patients died after more than 72 months (84-348 months). These patients, plus the 18 patients who remained alive (all alive for ≥96 months), with disease (14 patients), or free of metastatic disease (4 patients with regional lymph nodes metastasis removed with the primary tumor) were defined as iMPGL (4 late deaths + 18 alive). Due to the sample size, we performed only a univariate analysis [81], which showed differences in 3 variables when comparing aMPGL vs iMPGL (Table 11). The 3 variables were the presence of atypical mitoses (50% vs 0%; *P* = .029) and higher IHC-Ki-67 indices (median 5% [2.5%-8.5%] vs 0.6% [0.1%-6.1%]; *P* = .010) that were more frequent in aMPGL vs iMPGL, while lower concentrations of 24-hour urinary noradrenaline were observed in aMPGL vs iMPGL (median 84 mcg/24 hours [9-2763] vs 698.5 mcg/24 hours [170-5187]; *P* = .040) (see Table 11). Kaplan-Meier curves were generated for these 3 variables and there was a significant difference between the survival curves only regarding IHC–Ki-67 (<3% or ≥3%) (Fig. 6B).

**Table 11.** Characteristics associated with disease aggressiveness in metastatic paraganglioma

Variable	aMPGL(n = 8)	iMPGL (n = 22)	*P*
NAU (14-80 mcg/24 h)*^a^*	622.4 ± 1198.2 84 (9-2763) (5/8)	1635 ± 1938.9 698.5 (170-5187) (10/22)	.040*^d^*
Atypical mitotic figures*^b^*	50% (3/6)	0% (0/11)	.029*^d^*
Ki-67 (%)*^c^*	5.1 ± 2.4 5 (2.5-8.5) (5/8)	1.7 ± 2.0 0.6 (0.1-6.1) (13/22)	.010*^d^*
Ki-67 ≥ 3%*^c^*	80% (4/5)	23.1% (3/13)	.047*^d^*

Results expressed as mean ± SD, median (minimum-maximum), or percentage (n positive/n available).

Abbreviations: aMPGL, aggressive metastatic paraganglioma group; NAU, 24-hour urinary noradrenaline; iMPLG, indolent metastatic paraganglioma group.

^*a*^NAU results were available for 5 aMPGL patients and for 10 iMPGL patients.

^*b*^Atypical mitotic figures were assessed in 6 aMPGL tumors and in 11 iMPGL tumors.

^*c*^IHC-Ki-67 was assessed in 5 aMPGL tumors and in 13 iMPGL tumors.

^*d*^Significant *P* less than .05.

## Discussion

Of the total of initially selected patients, 13.3% (35/263) developed metastatic disease at a frequency similar to that described in the literature [12, 18, 82] and were classified as having MPGL. As metastases can occur a few months to several years after primary tumor surgery, it has not yet been established what disease-free interval could relatively safely classify a patient as having nonmetastatic disease [5, 10]. We defined this interval as 96 months or more, and we believe that a patient who does not develop metastasis after this long follow-up period is, with great probability, a carrier of NMPGL. Thus, 41.8% (110/263) of the patients were classified as having NMPGL, and 44.9% (118/263) were excluded mainly due to insufficient follow-up time. Therefore, we evaluated 35 MPGL and 110 NMPGL cases (see Fig. 1).

Many of the variables analyzed showed different expressions in the univariate analysis for MPGL vs NMPGL. Since we had a total of 35 positive outcomes, we selected 7 variables for the multivariable analysis. The variables were chosen according to their OR in the univariate analysis and when more than one histological variable representing the same histological phenomenon was available, one variable was chosen based on the its reproducibility and OR. Thus, the variables selected for the multivariable analysis were central or confluent necrosis, mitotic index more than 3 mitoses/10 HPF, high cellularity, tumor size of 8.1 cm or greater, PGLexAd, presence of PV in the *SDHB* gene, and extension to adipose tissue. Three histological variables (central or confluent necrosis, >3 mitoses/10 HPF, and extension to adipose tissue) and one nonhistological variable (extra-adrenal tumor location) remained independently related to the metastatic behavior of the tumor. The selected histological variables are present in the PASS score and received the maximum weight in it (weight = 2) [32]. They indicate rapid tumor growth (central or confluent necrosis), high cell proliferative index (>3 mitoses/10 HPF), and invasive tumor (extension to adipose tissue). The nonhistological variable (extra-adrenal location) has already been identified as a predictor of metastatic disease in several studies [12, 22, 23, 49, 52]. In the TNM staging system for tumor staging, extra-adrenal paraganglioma are classified as T3, regardless of size [5]. Although all PGLs have the same cellular origin, extra-adrenal paragangliomas, especially abdominal and pelvic tumors, more frequently present with more aggressive biological behavior, which may be related both to their genetic basis (eg, PV in the *SDHB* gene) and to other, not yet identified factors [12, 18].

The presence of PV in the *SDHB* gene was selected for multivariable analysis but did not prove to be an independent risk factor for MPGL. However, its association with the metastatic behavior of the tumor has been widely demonstrated in the literature [18, 28-31, 55, 83, 84]. We believe that this unexpected result found in this study was due to the insufficient number of patients with complete molecular investigation for genetic disease. Tumor size, a variable widely assessed to differentiate between MPGL and NMPGL, shows a controversial relationship with metastatic potential; it was positive in some studies [12, 16, 37, 43] and showed no importance in others [17, 32, 85, 86]. In the present study, even by adopting the cutoff value of 8.1 cm, this variable was not independently related to tumor behavior. Although not included in the multivariable analysis, 24-hour urinary noradrenaline was higher in the MPGL group. Catecholamine type produced by the tumor represents a nonhistological parameter that seems to relate to cellular differentiation; poor differentiated tumors may present with impairment of several enzymes involved in the synthesis of catecholamines leading to preferential synthesis of adrenaline precursors such as noradrenaline and dopamine [20, 33, 52]. Dopamine urinary concentrations were higher in MPGL (*P* = .017), with OR = 4.396 but with a 95% CI of 0.482 to 40.104. This could possibly be associated with limitations inherent to dopamine detection methods [87] since its metabolite, methoxytyramine, has been pointed out as a marker of metastatic disease [22]. Unfortunately, the assessment of this compound is not available in our service. Ki-67, which is widely used in assessing the metastatic potential of PGL [23, 44-47], was not included in the multivariable analysis, as it represents cellular proliferation already identified in histology as the mitotic index. The choice of more than 3 mitoses/10 HPF was based on its higher OR in the univariate analysis and on the fact that its analysis exempts the need for IHC. CHGB has been considered an inversely related factor to the metastatic potential of PGL [38, 59]. In our evaluation, this was not clearly demonstrated because, although it has high specificity for identifying NMPGL (85%), the AUC-ROC was small, which demonstrates the low efficiency of this variable in discriminating MPGL from NMPGL [81].

It is worth noting that the CART peptide, evaluated in IHC, was not useful in differentiating metastatic potential in PGL. We believed that this marker could be a possible predictor of malignant behavior in these tumors, as it was shown to be related to disease progression in PGLs [65, 66]. However, IHC-CART was weak in most PGLs and similar in MPGL vs NMPGL.

The 4 variables selected were assigned points according to their relative importance in the outcome (metastasis) (see Table 8). Based on these values we generated the PSPGL score, which was calculated only for tumors with results available for the 4 variables (78 tumors: 19 MPGL and 59 NMPGL). A PSPGL score of 24 or greater discriminated MPGL from the NMPGL patients with a sensitivity of 89.5%, specificity of 91.5%, VPP of 77.3%, and NPV of 96.4% (see Fig. 4 and Table 9). We calculated in the tumors in the present study, the PASS, GAPP, and PSPGL and compared the CI of these indices. We demonstrated that the 3 scores had similar sensitivity and accuracy, and PSPGL had greater specificity (see Table 9). Table 12 shows sensitivity, specificity, PPV, and NPV of PASS [32] and GAPP [33] original studies and PSPGL.

**Table 12.** Sensitivity, specificity, positive predictive value, and negative predictive value of the Pheochromocytoma of the Adrenal Gland Scaled Score*^c^*, Grading System for Adrenal Pheochromocytoma and Paraganglioma*^c^*, and Prognostic Score in Paragangliomas

Variables	PASS	GAPP	PSPGL
PGL (n)	100	163	**78** *^b^*
MPGL (n)	33*^a^*	40	**19**
Sensitivity	100% (33/33)	90% (36/40)	**89.5% (17/19)**
Specificity	75% (50/67)	87% (107/123)	**91.5% (54/59)**
NPV	100% (50/50)	96% (107/111)	**96.4% (54/56)**
PPV	66% (33/50)	69% (36/52)	**77.3% (17/22)**

Abbreviations: GAPP, Grading System for Adrenal Pheochromocytoma and Paraganglioma [33]; MPGL, metastatic paragangliomas; NPV, negative predictive value; PASS, Pheochromocytoma of the Adrenal Gland Scaled Score [32]; PGL, paragangliomas; PPV, positive predictive value; PSPGL, Prognostic Score in Paragangliomas.

^*a*^Seventeen of 50 patients did not present with metastatic disease.

^*b*^PSPGL was calculated only for tumors with available data for the 4 parameters included in the score.

^*c*^Results of comparison between PSPGL and PASS and GAPP original studies.

As previously discussed, the main issue with the classically used scores is the limitation regarding specificity and accuracy for predicting metastatic PGL (PPV) [40, 41]. PSPGL presented 91.5% specificity and 77.3% PPV, higher than those observed in the original studies of PASS and GAPP (see Table 12). We consider that the main advantage of the PSPGL is that it is derived from a smaller number of variables—only 4—which are generally available and easily reproducible. This will allow it to be more widely used because it is more accessible and will likely have less interobserver variability than classic scores.

When we analyzed the logistic regression curve of the PSPGL, we verified that the estimated probability of metastasis and the actual incidence of this occurrence are very similar, which reinforces the high capacity of this score in predicting metastatic behavior (see Fig. 5). According to the score achieved by tumors in the PSPGL score, patients can be classified regarding their risk of developing metastatic disease as follows: 1, very low risk (PSPGL = 0 points: probability ∼0%) and low risk (PSPGL = 19-20 points [extra-adrenal PGLs without any of the 3 histological variables or adrenal PGLs only with extension to adipose tissue]: probability ∼10%); 2, moderate risk (20 < PSPGL ≤ 39: probability of 10%-80%); and 3, high risk (PSPGL ≥ 40 points: probability >80%). PSPGL identified, with greater certainty, patients with low (<10%) and high (80%-100%) probability of developing metastases but did not clearly identify this probability in patients with intermediate scores (12 patients). Of these, 5 had NMPGL, and the evaluation of Ki-67 showed values of 0.1% to 0.8% in 3 patients, 1.3% and 3.6% in 2 patients (carriers of PV in *VHL*, which was present only in NMPGL patients). In the other 7 patients with MPGL, the evaluation of Ki-67 showed values of 0.1% to 3.7% in 4 patients with iMPGL and 5% to 6.2% in 3 patients with aMPGL, 2 of whom were carriers of PV in *SDHB.* Therefore, we suggest that in patients with intermediate PSPGL (20 < PSPGL ≤ 39), we should consider other factors for risk prediction such as Ki-67 and the presence of PV in genes that are associated or not with metastatic disease. Our findings do not allow for definitive conclusions on the time, frequency, and quality of monitoring of clinical, laboratory, and imaging data of patients with nonmetastatic disease at the time of surgery, based only on the PSPGL. However, we recommend that patients with high-risk tumors (PSPGL ≥ 40) be monitored preferably every 6 months in the first 4 years following surgery (mean time to appearance of metastasis = 44 months). If patients remain disease free, tests can then be performed annually during the next 4 years. If they continue to be disease free, these patients can undergo clinical examination and laboratory tests every 2 years for an extended period. The PSPGL also allows us to recommend that patients with very low or low risk be followed-up with annual clinical examination and biochemical tests, and imaging exams every 2 years. These patients can be considered nonmetastatic after 8 years of follow-up, but they must remain under observation. It is not possible to make any other more precise recommendation for patients with an intermediate risk (20 < PSPGL ≤ 39) based only on the PSPGL assessment, and in these cases, we recommend using other markers of metastatic (eg, Ki-67 ≥ 3%, PV in *SDHB*) or nonmetastatic disease (eg, PVs in *VHL*, *RET*, *TMEM127*). According to these markers that are not part of the PSPGL, patients should be monitored as low or high risk.

The identification of prognostic factors for MPGL is also a topic of great interest. As demonstrated in the survival curve of patients with MPGL, there are two types of tumor behavior, one more aggressive and responsible for short survival (aMPGL) and one more indolent that allows for long survival (iMPGL) (Fig. 6A). Studies related to the progression of metastatic tumors are difficult to conduct due to the rarity of PGLs and, mainly, of MPGL. Older age at diagnosis, male sex, synchronous metastases, and increased plasma concentrations of dopamine and methoxytyramine are factors that have been related to shorter survival in some studies [8, 9, 12, 14, 88, 89]. In the present study, the small number of tumors assessed (8 aMPGL vs 22 iMPGL) allowed the comparison among the several variables only by using univariate analysis. This analysis showed that 3 variables presented a positive correlation with poor prognosis: presence of atypical mitosis, Ki-67 of 3% or greater, and smaller concentrations of 24-hour urinary noradrenaline. Kaplan-Meier curves were generated for patients with MPGL taking into account these variables, and only Ki-67of 3% or greater was associated with shorter DSS. This result is consistent with the results of a multicenter European study that included 169 patients with metastatic disease that found IHC–Ki-67 of 2% or less was associated with better survival [8]. We found that survival at 8 years was approximately 90% and 38% in patients with tumors with IHC–Ki67 less than 3% and 3% or greater, respectively (Fig. 6B). Synchrony and shorter time elapsed between surgery and detection of the metastasis have been studied as worse prognostic factors in MPGL [8, 14, 88, 89]. In this study, it was not possible to establish a relationship between these variables and prognosis, and this may be attributed to the small number of patients. The presence of PV in the *SDHB* gene has an already established relationship with metastatic potential but does not seem to be related to shorter survival [8, 14].

The main limitations of this study include its sample size, which, although numerically important, if we consider a single study center, was still small, especially the absolute number of patients with metastatic disease; difficulties in data collection inherent to retrospective studies; impossibility of obtaining data related to genetic diagnosis due to the unavailability of molecular assessments prior to 2014, and the fact that the current assessment, although systematic, does not reach all genes involved in the pathogenesis of PGLs; tumor functionality-type assessments were impaired prior to 2012 because free and fractionated metanephrine assessments were not available; and finally, our score has not yet been internally or externally validated.

In summary, we proposed a prognostic score for PGLs, the PSPGL, which includes a nonhistological variable (extra-adrenal location) and 3 histological variables (central or confluent necrosis, mitotic index >3 mitoses/10 HPF, and extension to adipose tissue), all easily assessed. The PSPGL showed a performance similar to the PASS and GAPP but with higher specificity. The PSPGL score showed good capacity in predicting low and high risk of metastases. Genetic diagnosis and the Ki-67 index can be auxiliary tools in predicting risk in patients with intermediate scores. IHC–Ki-67 greater than or equal to 3% was shown to be a predictor of worse prognosis in MPGL.

## Data Availability

Original data generated and analyzed during this study are included in this published article or in the data repositories listed in “References.”

## Abbreviations

β coef: β coefficient
aMPGL: aggressive metastatic paraganglioma
AUC: area under the curve
CART: cocaine- and amphetamine-regulated transcript
CHGA: chromogranin A
CHGB: chromogranin B
CT: computed tomography
DSS: disease-specific survival
GAPP: Grading System for Adrenal Pheochromocytoma and Paraganglioma
IHC: immunohistochemistry
iMPGL: indolent metastatic paraganglioma
MPGL: metastatic paraganglioma
NPV: negative predictive value
OR: odds ratio
PASS: Pheochromocytoma of the Adrenal Gland Scaled Score
PET: positron emission tomography
PI: positivity index
PGL: paraganglioma
PGLAd: adrenal paragangliomas
PGLexAd: extra-adrenal paragangliomas
PPV: positive predictive value
PSPGL: Prognostic Score in Paragangliomas
PV: pathogenic variant
ROC: receiver operating characteristic
SDHB: subunit B of succinate dehydrogenase
VHL: von Hippel-Lindau disease

## References

[1] Mete O, Asa SL, Gill AJ, Kimura N, de Krijger RR, Tischler A. Overview of the 2022 WHO classification of paragangliomas and pheochromocytomas. Endocr Pathol. 2022;33(1):90‐114. 35285002 10.1007/s12022-022-09704-6

[2] Lenders JWM, Kerstens MN, Amar L, et al Genetics, diagnosis, management and future directions of research of phaeochromocytoma and paraganglioma: a position statement and consensus of the Working Group on Endocrine Hypertension of the European Society of Hypertension. J Hypertens. 2020;38(8):1443‐1456. 32412940 10.1097/HJH.0000000000002438 PMC7486815

[3] Lenders JWM, Duh QY, Eisenhofer G, et al Pheochromocytoma and paraganglioma: an endocrine society clinical practice guideline. J Clin Endocrinol Metab. 2014;99(6):1915‐1942. 24893135 10.1210/jc.2014-1498

[4] Al Subhi AR, Boyle V, Elston MS. Systematic review: incidence of pheochromocytoma and paraganglioma over 70 years. J Endocr Soc. 2022;6(9):bvac105. 35919261 10.1210/jendso/bvac105 PMC9334688

[5] Lloyd RV, Osamura RY, Klöppel G, Rosai J, World Health Organization, International Agency for Research on Cancer. WHO Classification of Tumours of Endocrine Organs. 4th ed. International Agency for Research on Cancer (IARC); 2017:355.

[6] Baudin E, Habra MA, Deschamps F, et al Therapy of endocrine disease: treatment of malignant pheochromocytoma and paraganglioma. Eur J Endocrinol. 2014;171(3):R111‐R122. 24891137 10.1530/EJE-14-0113

[7] Scholz T, Eisenhofer G, Pacak K, Dralle H, Lehnert H. Clinical review: current treatment of malignant pheochromocytoma. J Clin Endocrinol Metab. 2007;92(4):1217‐1225. 17284633 10.1210/jc.2006-1544

[8] Hescot S, Curras-Freixes M, Deutschbein T, et al Prognosis of malignant pheochromocytoma and paraganglioma (MAPP-Prono Study): a European Network for the Study of Adrenal Tumors Retrospective Study. J Clin Endocrinol Metab. 2019;104(6):2367‐2374. 30715419 10.1210/jc.2018-01968

[9] Hamidi O, Young WF, Iñiguez-Ariza NM, et al Malignant pheochromocytoma and paraganglioma: 272 patients over 55 years. J Clin Endocrinol Metab. 2017;102(9):3296‐3305. 28605453 10.1210/jc.2017-00992 PMC5587061

[10] Plouin PF, Amar L, Dekkers OM, et al European Society of Endocrinology Clinical Practice Guideline for long-term follow-up of patients operated on for a phaeochromocytoma or a paraganglioma. Eur J Endocrinol. 2016;174(5):G1‐G10. 27048283 10.1530/EJE-16-0033

[11] Roman-Gonzalez A, Jimenez C. Malignant pheochromocytoma-paraganglioma: pathogenesis, TNM staging, and current clinical trials. Curr Opin Endocrinol Diabetes Obes. 2017;24(3):174‐183. 28234804 10.1097/MED.0000000000000330

[12] Ayala-Ramirez M, Feng L, Johnson MM, et al Clinical risk factors for malignancy and overall survival in patients with pheochromocytomas and sympathetic paragangliomas: primary tumor size and primary tumor location as prognostic indicators. J Clin Endocrinol Metab. 2011;96(3):717‐725. 21190975 10.1210/jc.2010-1946

[13] Granberg D, Juhlin CC, Falhammar H. Metastatic pheochromocytomas and abdominal paragangliomas. J Clin Endocrinol Metab. 2021;106(5):e1937‐e1952. 33462603 10.1210/clinem/dgaa982 PMC8063253

[14] Hamidi O, Young WF, Gruber L, et al Outcomes of patients with metastatic phaeochromocytoma and paraganglioma: a systematic review and meta-analysis. Clin Endocrinol (Oxf). 2017;87(5):440‐450. 28746746 10.1111/cen.13434 PMC5854189

[15] Fagundes GFC, Almeida MQ. Perioperative management of pheochromocytomas and sympathetic paragangliomas. J Endocr Soc. 2022;6(2):bvac004. 35128297 10.1210/jendso/bvac004 PMC8807163

[16] Zelinka T, Musil Z, Dušková J, et al Metastatic pheochromocytoma: does the size and age matter? Eur J Clin Invest. 2011;41(10):1121‐1128. 21692797 10.1111/j.1365-2362.2011.02518.x PMC3170415

[17] Kim KY, Kim JH, Hong AR, et al Disentangling of malignancy from benign pheochromocytomas/paragangliomas. PLoS One. 2016;11(12):e0168413. 27992508 10.1371/journal.pone.0168413 PMC5161476

[18] Turkova H, Prodanov T, Maly M, et al Characteristics and outcomes of metastatic SDHB and sporadic pheochromocytoma/paraganglioma: an national institutes of health study. Endocr Pract. 2016;22(3):302‐314. 26523625 10.4158/EP15725.OR PMC7473461

[19] Dhir M, Li W, Hogg ME, et al Clinical predictors of malignancy in patients with pheochromocytoma and paraganglioma. Ann Surg Oncol. 2017;24(12):3624‐3630. 28884434 10.1245/s10434-017-6074-1

[20] van der Harst E, de Herder WW, de Krijger RR, et al The value of plasma markers for the clinical behaviour of phaeochromocytomas. Eur J Endocrinol. 2002;147(1):85‐94. 12088924 10.1530/eje.0.1470085

[21] Plouin PF, Chatellier G, Fofol I, Corvol P. Tumor recurrence and hypertension persistence after successful pheochromocytoma operation. Hypertension. 1997;29(5):1133‐1139. 9149678 10.1161/01.hyp.29.5.1133

[22] Eisenhofer G, Lenders JW, Siegert G, et al Plasma methoxytyramine: a novel biomarker of metastatic pheochromocytoma and paraganglioma in relation to established risk factors of tumour size, location and SDHB mutation status. Eur J Cancer. 2012;48(11):1739‐1749. 22036874 10.1016/j.ejca.2011.07.016 PMC3372624

[23] van der Harst E, Bruining HA, Jaap Bonjer H, et al Proliferative index in phaeochromocytomas: does it predict the occurrence of metastases? J Pathol. 2000;191(2):175‐180. 10861578 10.1002/(SICI)1096-9896(200006)191:2<175::AID-PATH615>3.0.CO;2-Z

[24] Nicolas M, Dahia P. Predictors of outcome in phaeochromocytomas and paragangliomas. F1000Res. 2017;6:2160. 29333259 10.12688/f1000research.12419.1 PMC5749134

[25] Assadipour Y, Sadowski SM, Alimchandani M, et al SDHB mutation status and tumor size but not tumor grade are important predictors of clinical outcome in pheochromocytoma and abdominal paraganglioma. Surgery. 2017;161(1):230‐239. 27839933 10.1016/j.surg.2016.05.050 PMC5164946

[26] Gimenez-Roqueplo AP, Favier J, Rustin P, et al Mutations in the SDHB gene are associated with extra-adrenal and/or malignant phaeochromocytomas. Cancer Res. 2003;63(17):5615‐5621. 14500403

[27] Amar L, Baudin E, Burnichon N, et al Succinate dehydrogenase B gene mutations predict survival in patients with malignant pheochromocytomas or paragangliomas. J Clin Endocrinol Metab. 2007;92(10):3822‐3828. 17652212 10.1210/jc.2007-0709

[28] Brouwers FM, Eisenhofer G, Tao JJ, et al High frequency of SDHB germline mutations in patients with malignant catecholamine-producing paragangliomas: implications for genetic testing. J Clin Endocrinol Metab. 2006;91(11):4505‐4509. 16912137 10.1210/jc.2006-0423

[29] Pasini B, Stratakis CA. SDH mutations in tumorigenesis and inherited endocrine tumours: lesson from the phaeochromocytoma-paraganglioma syndromes. J Intern Med. 2009;266(1):19‐42. 19522823 10.1111/j.1365-2796.2009.02111.x PMC3163304

[30] King KS, Prodanov T, Kantorovich V, et al Metastatic pheochromocytoma/paraganglioma related to primary tumor development in childhood or adolescence: significant link to SDHB mutations. J Clin Oncol. 2011;29(31):4137‐4142. 21969497 10.1200/JCO.2011.34.6353 PMC3208535

[31] van Hulsteijn LT, Dekkers OM, Hes FJ, Smit JW, Corssmit EP. Risk of malignant paraganglioma in SDHB-mutation and SDHD-mutation carriers: a systematic review and meta-analysis. J Med Genet. 2012;49(12):768‐776. 23099648 10.1136/jmedgenet-2012-101192

[32] Thompson LD . Pheochromocytoma of the adrenal gland scaled score (PASS) to separate benign from malignant neoplasms: a clinicopathologic and immunophenotypic study of 100 cases. Am J Surg Pathol. 2002;26(5):551‐566. 11979086 10.1097/00000478-200205000-00002

[33] Kimura N, Watanabe T, Noshiro T, Shizawa S, Miura Y. Histological grading of adrenal and extra-adrenal pheochromocytomas and relationship to prognosis: a clinicopathological analysis of 116 adrenal pheochromocytomas and 30 extra-adrenal sympathetic paragangliomas including 38 malignant tumors. Endocr Pathol. 2005;16(1):23‐32. 16000843 10.1385/ep:16:1:023

[34] August C, August K, Schroeder S, et al CGH and CD 44/MIB-1 immunohistochemistry are helpful to distinguish metastasized from nonmetastasized sporadic pheochromocytomas. Mod Pathol. 2004;17(9):1119‐1128. 15167935 10.1038/modpathol.3800160

[35] Kulkarni MM, Khandeparkar SG, Deshmukh SD, et al Risk stratification in paragangliomas with PASS (pheochromocytoma of the adrenal gland scaled score) and immunohistochemical markers. J Clin Diagn Res. 2016;10(9):EC01‐EEC4. 10.7860/JCDR/2016/20565.8419 PMC5071941 27790441

[36] Koh JM, Ahn SH, Kim H, et al Validation of pathological grading systems for predicting metastatic potential in pheochromocytoma and paraganglioma. PLoS One. 2017;12(11):e0187398. 29117221 10.1371/journal.pone.0187398 PMC5678867

[37] Pierre C, Agopiantz M, Brunaud L, et al COPPS, a composite score integrating pathological features, PS100 and SDHB losses, predicts the risk of metastasis and progression-free survival in pheochromocytomas/paragangliomas. Virchows Arch. 2019;474(6):721‐734. 30868297 10.1007/s00428-019-02553-5

[38] Stenman A, Svahn F, Hojjat-Farsangi M, et al Molecular profiling of pheochromocytoma and abdominal paraganglioma stratified by the PASS algorithm reveals chromogranin B as associated with histologic prediction of malignant behavior. Am J Surg Pathol. 2019;43(3):409‐421. 30451732 10.1097/PAS.0000000000001190

[39] Wu D, Tischler AS, Lloyd RV, et al Observer variation in the application of the pheochromocytoma of the adrenal gland scaled score. Am J Surg Pathol. 2009;33(4):599‐608. 19145205 10.1097/PAS.0b013e318190d12e

[40] Wachtel H, Hutchens T, Baraban E, et al Predicting metastatic potential in pheochromocytoma and paraganglioma: a comparison of PASS and GAPP scoring systems. J Clin Endocrinol Metab. 2020;105(12):e4661‐e4670. 32877928 10.1210/clinem/dgaa608 PMC7553245

[41] Stenman A, Zedenius J, Juhlin CC. The value of histological algorithms to predict the malignancy potential of pheochromocytomas and abdominal paragangliomas-a meta-analysis and systematic review of the literature. Cancers (Basel). 2019;11(2):225. 30769931 10.3390/cancers11020225 PMC6406721

[42] Kimura N, Takayanagi R, Takizawa N, et al Pathological grading for predicting metastasis in phaeochromocytoma and paraganglioma. Endocr Relat Cancer. 2014;21(3):405‐414. 24521857 10.1530/ERC-13-0494

[43] Cho YY, Kwak MK, Lee SE, et al A clinical prediction model to estimate the metastatic potential of pheochromocytoma/paraganglioma: ASES score. Surgery. 2018;164(3):511‐517. 29929757 10.1016/j.surg.2018.05.001

[44] Gupta D, Shidham V, Holden J, Layfield L. Prognostic value of immunohistochemical expression of topoisomerase alpha II, MIB-1, p53, E-cadherin, retinoblastoma gene protein product, and HER-2/neu in adrenal and extra-adrenal pheochromocytomas. Appl Immunohistochem Mol Morphol. 2000;8(4):267‐274. 11127918

[45] Ohji H, Sasagawa I, Iciyanagi O, Suzuki Y, Nakada T. Tumour angiogenesis and Ki-67 expression in phaeochromocytoma. BJU Int. 2001;87(4):381‐385. 11251535 10.1046/j.1464-410x.2001.00102.x

[46] Brown HM, Komorowski RA, Wilson SD, Demeure MJ, Zhu YR. Predicting metastasis of pheochromocytomas using DNA flow cytometry and immunohistochemical markers of cell proliferation: a positive correlation between MIB-1 staining and malignant tumor behavior. Cancer. 1999;86(8):1583‐1589. 10526289

[47] Nagura S, Katoh R, Kawaoi A, Kobayashi M, Obara T, Omata K. Immunohistochemical estimations of growth activity to predict biological behavior of pheochromocytomas. Mod Pathol. 1999;12(12):1107‐1111. 10619262

[48] Häyry V, Salmenkivi K, Arola J, Heikkilä P, Haglund C, Sariola H. High frequency of SNAIL-expressing cells confirms and predicts metastatic potential of phaeochromocytoma. Endocr Relat Cancer. 2009;16(4):1211‐1218. 19641025 10.1677/ERC-09-0049

[49] Korevaar TI, Grossman AB. Pheochromocytomas and paragangliomas: assessment of malignant potential. Endocrine. 2011;40(3):354‐365. 22038451 10.1007/s12020-011-9545-3

[50] Xu Y, Qi Y, Rui W, et al Expression and diagnostic relevance of heat shock protein 90 and signal transducer and activator of transcription 3 in malignant pheochromocytoma. J Clin Pathol. 2013;66(4):286‐290. 23322822 10.1136/jclinpath-2012-201134

[51] Leijon H, Salmenkivi K, Heiskanen I, et al Hur in pheochromocytomas and paragangliomas—overexpression in verified malignant tumors. APMIS. 2016;124(9):757‐763. 27357268 10.1111/apm.12571

[52] Feng F, Zhu Y, Wang X, et al Predictive factors for malignant pheochromocytoma: analysis of 136 patients. J Urol. 2011;185(5):1583‐1590. 21419457 10.1016/j.juro.2010.12.050

[53] Pinato DJ, Ramachandran R, Toussi ST, et al Immunohistochemical markers of the hypoxic response can identify malignancy in phaeochromocytomas and paragangliomas and optimize the detection of tumours with VHL germline mutations. Br J Cancer. 2013;108(2):429‐437. 23257898 10.1038/bjc.2012.538 PMC3566818

[54] Fischer A, Kloos S, Maccio U, et al Metastatic pheochromocytoma and paraganglioma: somatostatin receptor 2 expression, genetics, and therapeutic responses. J Clin Endocrinol Metab. 2023;108(10):2676‐2685. 36946182 10.1210/clinem/dgad166 PMC10505550

[55] Fishbein L, Leshchiner I, Walter V, et al Comprehensive molecular characterization of pheochromocytoma and paraganglioma. Cancer Cell. 2017;31(2):181‐193. 28162975 10.1016/j.ccell.2017.01.001 PMC5643159

[56] Fishbein L, Khare S, Wubbenhorst B, et al Whole-exome sequencing identifies somatic ATRX mutations in pheochromocytomas and paragangliomas. Nat Commun. 2015;6(1):6140. 25608029 10.1038/ncomms7140 PMC4302757

[57] Job S, Draskovic I, Burnichon N, et al Telomerase activation and ATRX mutations are independent risk factors for metastatic pheochromocytoma and paraganglioma. Clin Cancer Res. 2019;25(2):760‐770. 30301828 10.1158/1078-0432.CCR-18-0139

[58] Boltze C, Mundschenk J, Unger N, et al Expression profile of the telomeric complex discriminates between benign and malignant pheochromocytoma. J Clin Endocrinol Metab. 2003;88(9):4280‐4286. 12970299 10.1210/jc.2002-021299

[59] Wang Y, Chen D, Pang Y, Xu X, Guan X, Liu L. Value of immunohistochemical expression of apelin, succinate dehydrogenase B, chromogranin B, human epidermal growth factor receptor-2, contactin 4, and succinyl-CoA synthetase subunit beta in differentiating metastatic from non-metastatic pheochromocytoma and paraganglioma. Front Endocrinol (Lausanne). 2022;13:882906. 35574028 10.3389/fendo.2022.882906 PMC9096168

[60] Lloyd RV, Blaivas M, Wilson BS. Distribution of chromogranin and S100 protein in normal and abnormal adrenal medullary tissues. Arch Pathol Lab Med. 1985;109(7):633‐635. 3839362

[61] Unger P, Hoffman K, Pertsemlidis D, Thung S, Wolfe D, Kaneko M. S100 protein-positive sustentacular cells in malignant and locally aggressive adrenal pheochromocytomas. Arch Pathol Lab Med. 1991;115(5):484‐487. 1673596

[62] Liu TH, Chen YJ, Wu SF, et al [Distinction between benign and malignant pheochromocytomas]. Zhonghua Bing Li Xue Za Zhi. 2004;33(3):198‐202. 15256107

[63] de Wailly P, Oragano L, Radé F, et al Malignant pheochromocytoma: new malignancy criteria. Langenbecks Arch Surg. 2012;397(2):239‐246. 22069042 10.1007/s00423-011-0850-3

[64] Douglass J, McKinzie AA, Couceyro P. PCR differential display identifies a rat brain mRNA that is transcriptionally regulated by cocaine and amphetamine. J Neurosci. 1995;15(3 Pt 2):2471‐2481. 7891182 10.1523/JNEUROSCI.15-03-02471.1995 PMC6578117

[65] Bech P, Winstanley V, Murphy KG, et al Elevated cocaine- and amphetamine-regulated transcript immunoreactivity in the circulation of patients with neuroendocrine malignancy. J Clin Endocrinol Metab. 2008;93(4):1246‐1253. 18211969 10.1210/jc.2007-1946 PMC2729185

[66] Ramachandran R, Bech P, Murphy KG, et al Comparison of the utility of cocaine- and amphetamine-regulated transcript (CART), chromogranin A, and chromogranin B in neuroendocrine tumor diagnosis and assessment of disease progression. J Clin Endocrinol Metab. 2015;100(4):1520‐1528. 25664601 10.1210/jc.2014-3640

[67] Bausch B, Schiavi F, Ni Y, et al Clinical characterization of the pheochromocytoma and paraganglioma susceptibility genes SDHA, TMEM127, MAX, and SDHAF2 for gene-informed prevention. JAMA Oncol. 2017;3(9):1204‐1212. 28384794 10.1001/jamaoncol.2017.0223 PMC5824290

[68] Castro-Vega LJ, Letouzé E, Burnichon N, et al Multi-omics analysis defines core genomic alterations in pheochromocytomas and paragangliomas. Nat Commun. 2015;6(1):6044. 25625332 10.1038/ncomms7044 PMC4354166

[69] Letouzé E, Martinelli C, Loriot C, et al SDH mutations establish a hypermethylator phenotype in paraganglioma. Cancer Cell. 2013;23(6):739‐752. 23707781 10.1016/j.ccr.2013.04.018

[70] Toledo RA, Qin Y, Cheng ZM, et al Recurrent mutations of chromatin-remodeling genes and kinase receptors in pheochromocytomas and paragangliomas. Clin Cancer Res. 2016;22(9):2301‐2310. 26700204 10.1158/1078-0432.CCR-15-1841 PMC4854762

[71] Welander J, Andreasson A, Juhlin CC, et al Rare germline mutations identified by targeted next-generation sequencing of susceptibility genes in pheochromocytoma and paraganglioma. J Clin Endocrinol Metab. 2014;99(7):E1352‐E1360. 24694336 10.1210/jc.2013-4375 PMC5393486

[72] Toledo RA, Burnichon N, Cascon A, et al Consensus statement on next-generation-sequencing-based diagnostic testing of hereditary phaeochromocytomas and paragangliomas. Nat Rev Endocrinol. 2017;13(4):233‐247. 27857127 10.1038/nrendo.2016.185

[73] Bankhead P, Loughrey MB, Fernández JA, et al Qupath: open source software for digital pathology image analysis. Sci Rep. 2017;7(1):16878. 29203879 10.1038/s41598-017-17204-5 PMC5715110

[74] Li ML, Fitzgerald PA, Price DC, Norton JA. Iatrogenic pheochromocytomatosis: a previously unreported result of laparoscopic adrenalectomy. Surgery. 2001;130(6):1072‐1077. 11742341 10.1067/msy.2001.118373

[75] Yu R, Sharaga D, Donner C, Palma Diaz MF, Livhits MJ, Yeh MW. Pheochromocytomatosis associated with a novel TMEM127 mutation. Endocrinol Diabetes Metab Case Rep. 2017;2017:17-0026. 10.1530/EDM-17-0026 PMC5445434 28567294

[76] Auerbach MS, Livhits MJ, Yu R. Pheochromocytomatosis treated with peptide receptor radionuclide therapy. Clin Nucl Med. 2022;47(3):e276‐e278. 35020659 10.1097/RLU.0000000000003973

[77] Robledo AB, Ponce Marco JL, Ibáñez TB, Meseguer Anastasio MF, Gómez-Gavara C. Pheochromocytomatosis: a risk after pheochromocytoma surgery. Am Surg. 2010;76(8):122‐124. 28958230

[78] Mehta HB, Mehta V, Girman CJ, Adhikari D, Johnson ML. Regression coefficient-based scoring system should be used to assign weights to the risk index. J Clin Epidemiol. 2016;79:22‐28. 27181564 10.1016/j.jclinepi.2016.03.031

[79] Moons KG, Harrell FE, Steyerberg EW. Should scoring rules be based on odds ratios or regression coefficients? J Clin Epidemiol. 2002;55(10):1054‐1055. 12464384 10.1016/s0895-4356(02)00453-5

[80] Youden WJ . Index for rating diagnostic tests. Cancer. 1950;3(1):32‐35. 15405679 10.1002/1097-0142(1950)3:1<32::aid-cncr2820030106>3.0.co;2-3

[81] Iguchi D . Supplemental data from Identification of predictors of metastatic potential in paragangliomas to develop a prognostic score (PSPGL). Published April 2024. Accessed April 17, 2024. 10.48472/deposita/GHPYDF. PMC11112433 38799767

[82] Patel D, Phay JE, Yen TWF, et al Update on pheochromocytoma and paraganglioma from the SSO endocrine/head and neck disease-site work group. Part 1 of 2: advances in pathogenesis and diagnosis of pheochromocytoma and paraganglioma. Ann Surg Oncol. 2020;27(5):1329‐1337. 32112212 10.1245/s10434-020-08220-3 PMC8655649

[83] Crona J, Lamarca A, Ghosal S, Welin S, Skogseid B, Pacak K. Genotype-phenotype correlations in pheochromocytoma and paraganglioma: a systematic review and individual patient meta-analysis. Endocr Relat Cancer. 2019;26(5):539‐550. 30893643 10.1530/ERC-19-0024 PMC6717695

[84] Fishbein L, Nathanson KL. Pheochromocytoma and paraganglioma: understanding the complexities of the genetic background. Cancer Genet. 2012;205(1-2):1‐11. 22429592 10.1016/j.cancergen.2012.01.009 PMC3311650

[85] Strong VE, Kennedy T, Al-Ahmadie H, et al Prognostic indicators of malignancy in adrenal pheochromocytomas: clinical, histopathologic, and cell cycle/apoptosis gene expression analysis. Surgery. 2008;143(6):759‐768. 18549892 10.1016/j.surg.2008.02.007

[86] Agarwal A, Mehrotra PK, Jain M, et al Size of the tumor and pheochromocytoma of the adrenal gland scaled score (PASS): can they predict malignancy? World J Surg. 2010;34(12):3022‐3028. 20703467 10.1007/s00268-010-0744-5

[87] Eisenhofer G, Pamporaki C, Lenders JWM. Biochemical assessment of pheochromocytoma and paraganglioma. Endocr Rev. 2023;44(5):862‐909. 36996131 10.1210/endrev/bnad011

[88] Pamporaki C, Prodanov T, Meuter L, et al Determinants of disease-specific survival in patients with and without metastatic pheochromocytoma and paraganglioma. Eur J Cancer. 2022;169:32‐41. 35500459 10.1016/j.ejca.2022.03.032

[89] Choi YM, Sung TY, Kim WG, et al Clinical course and prognostic factors in patients with malignant pheochromocytoma and paraganglioma: a single institution experience. J Surg Oncol. 2015;112(8):815‐821. 26464058 10.1002/jso.24063

